# Hippurate as a metabolomic marker of gut microbiome diversity: Modulation by diet and relationship to metabolic syndrome

**DOI:** 10.1038/s41598-017-13722-4

**Published:** 2017-10-20

**Authors:** Tess Pallister, Matthew A. Jackson, Tiphaine C. Martin, Jonas Zierer, Amy Jennings, Robert P. Mohney, Alexander MacGregor, Claire J. Steves, Aedin Cassidy, Tim D. Spector, Cristina Menni

**Affiliations:** 1 Department of Twin Research and Genetic Epidemiology, King’s College London, London, SE1 7EH UK; 2 Institute of Bioinformatics and Systems Biology, Helmholtz Zentrum München, Neuherberg, Germany; 3 Department of Nutrition & Preventive Medicine, Norwich Medical School, University of East Anglia, Norwich, UK; 4 Metabolon Inc., Research Triangle Park, NC 27709 USA

## Abstract

Reduced gut microbiome diversity is associated with multiple disorders including metabolic syndrome (MetS) features, though metabolomic markers have not been investigated. Our objective was to identify blood metabolite markers of gut microbiome diversity, and explore their relationship with dietary intake and MetS. We examined associations between Shannon diversity and 292 metabolites profiled by the untargeted metabolomics provider Metabolon Inc. in 1529 females from TwinsUK using linear regressions adjusting for confounders and multiple testing (Bonferroni: *P* < 1.71 × 10^−4^). We replicated the top results in an independent sample of 420 individuals as well as discordant identical twin pairs and explored associations with self-reported intakes of 20 food groups. Longitudinal changes in circulating levels of the top metabolite, were examined for their association with food intake at baseline and with MetS at endpoint. Five metabolites were associated with microbiome diversity and replicated in the independent sample. Higher intakes of fruit and whole grains were associated with higher levels of hippurate cross-sectionally and longitudinally. An increasing hippurate trend was associated with reduced odds of having MetS (OR: 0.795[0.082]; *P* = 0.026). These data add further weight to the key role of the microbiome as a potential mediator of the impact of dietary intake on metabolic status and health.

## Introduction

The diversity of bacteria in the human gut, both in term of the number of different microbes and the comparative evenness of their abundances, is associated with higher abundance of beneficial bacteria and is emerging as an important indicator of health^1–6^. Lower alpha-diversity (intra-individual diversity) is suggestive of dysbiosis (microbial imbalance) and has been associated with metabolic syndrome features^6^.

Microbes transform food- and host-derived metabolites, such as bile acids and fibre^7^, and polyphenols^8^. The profound contribution of the gut microbiome to metabolism has been shown in conventional versus germ-free mice, where conventional mice exhibited elevated blood levels of indole-containing compounds (e.g. indoxyl sulfate and indole-3-propionic acid), serotonin, sulfated compounds (e.g. phenyl and *p*-cresol sulfate), and glycine-conjugated compounds (hippuric acid, cinnamoylglycine and phenylpropionylglycine)^9^. Many of the above metabolites are food-derived; therefore, merging microbiome and metabolomics approaches with studies which capture habitual intake is the logical next step for improving our understanding of the complex interplay between diet, the microbiome and metabolic disease.

To date there are relatively few short-term human dietary intervention studies incorporating the microbiome and metabolome. It has been shown that daily consumption of 40 g of dark chocolate for 2 weeks altered urinary output of gut microbial metabolites, increasing hippurate and methylamines, and reducing p-cresol sulfate^10^. Moreover, a recent randomized controlled pilot study showed feeding 30 g/d of heat-stabilized rice bran for 28 days increased abundance of 11 operational taxonomic units (OTUs), and elevated faecal levels of secondary bile acids and metabolites derived from microbial modifications of plant-derived components^11^.

To our knowledge, the role of a diverse gut microbiome in humans as a potential mediator of the impact of dietary intake on metabolic status and health has not been robustly addressed. Therefore, the aims of this study are: to (i) identify blood metabolites correlated with gut microbiome diversity, (ii) examine the impact of food intake on these metabolites and to, (iii) examine if longitudinal changes in these metabolites are predictive of future development of the metabolic syndrome.

## Results

Supplementary Table S1 provides the study population characteristics and subject numbers.

### Microbiome diversity metabolomics associations

Eight metabolites significantly correlated with Shannon diversity in the discovery sample after adjusting for multiple testing (Table 1). These include hippurate, p-cresol sulfate, phenylacetylglutamine, 4-ethylphenolsulfate, indolepropionate and 3-phenylpropionate which were positively associated; and hyodeoxycholate and phenol sulphate which were negatively associated. Five metabolites were validated in the replication sample (Table 1). These include hippurate, p-cresol sulfate, phenylacetylglutamine, 3-phenylpropionate, and hyodeoxycholate.

**Table 1:** Metabolites associated with Shannon diversity in the discovery sample (following backward stepwise linear regression) and in the validation sample^1^.

Metabolite	Super-pathway	Sub-pathway	Discovery (*n* = 1529)		Validation (*n* = 420)^2^	
			beta (SE)	*P*	beta (SE)	*P*
Hippurate	Xenobiotics	Benzoate metabolism	0.230 (0.040)	3.72 × 10^−8^	0.238 (0.072)	0.001*
p-cresol sulfate	Amino acid	Phenylalanine & tyrosine metabolism	0.200 (0.040)	9.90 × 10^−8^	0.179 (0.063)	0.005*
phenol sulfate	Amino acid	Phenylalanine & tyrosine metabolism	−0.200 (0.040)	5.82 × 10^−7^	−0.121 (0.063)	0.055
Phenylacetylglutamine	Amino acid	Phenylalanine & tyrosine metabolism	0.180 (0.040)	5.21 × 10^−6^	0.195 (0.062)	0.002*
3-phenylpropionate (hydrocinnamate)	Amino acid	Phenylalanine & tyrosine metabolism	0.160 (0.040)	3.43 × 10^−5^	0.185 (0.084)	0.028*
4-ethylphenylsulfate	Xenobiotics	Benzoate metabolism	0.190 (0.050)	5.12 × 10^−5^	0.062 (0.081)	0.441
Hyodeoxycholate	Lipid	Bile acid metabolism	−0.190 (0.050)	8.66 × 10^−5^	−0.215 (0.089)	0.016*
Indolepropionate	Amino acid	Tryptophan metabolism	0.140 (0.040)	9.20 × 10^−5^	0.093 (0.083)	0.262

^*^Statistically significant: *P* < 0.05.

^1^A linear regression was performed using Shannon diversity to predict levels of 292 metabolites adjusting for age, BMI, batch effects (and sex in the validation) and family relatedness.

^2^Statistically significant (*P* < 1.71 × 10^−4^) associations from the discovery group were validated in the validation group.

Higher circulating levels of the benzoate metabolite, hippurate, were also associated with higher fruit (0.012[0.002]; *P* = 7.36 × 10^−8^) and whole grains intake (0.013[0.003]; *P* = 2.05 × 10^−5^). Another benzoate metabolite, 3-phenylpropionate, was also positively associated with whole grain (0.018[0.004]; *P* = 2.71 × 10^−6^) and fruit intake (0.010[0.002]; *P* = 2.45 × 10^−5^) while higher levels were inversely associated with lower fried and fast food intake (−0.045[0.009]; *P* = 5.63 × 10^−7^). Hippurate and 3-phenylpropionate levels were correlated (*r* = 0.51; *P* < 0.001), although summing the two metabolites did not improve their association with Shannon diversity (hippurate R^2^: 0.0258; 3-phenylpropionate R^2^: 0.0122; and combined R^2^: 0.0236), therefore for the remainder of the analysis we focused on hippurate.

### Food intakes predict longitudinal hippurate trajectories

In a subsample of the discovery group higher baseline intakes of whole grains (1.70 × 10^−4^[3.84 × 10^−5^]; *P* = 9.54 × 10^−6^), coffee (1.03 × 10^−4^[2.82 × 10^−5^]; *P* = 2.73 × 10^−4^) and fruit (8.43 × 10^−5^[2.71 × 10^−5^]; *P* = 1.89 × 10^−3^) significantly (*P* < 0.0025) predicted increasing hippurate trends. All food associations were independent, remaining significant in a multivariate linear regression model and together accounted for 5.3% of the variance in hippurate trend. We calculated a hippurate diet score (computed as the quartile sum of these three food intakes) in the discovery sample (hippurate association: 0.089[0.012]; *P* = 1.13 × 10^−13^, and Shannon diversity association: 0.031[0.009]; *P* = 5.76 × 10^−4^) and validated it in the validation sample against hippurate levels (0.089[0.024]; *P* = 3.21 × 10^−4^), and Shannon diversity (0.040[0.019]; *P* = 0.035) (independently of hippurate). The hippurate diet score was moderately heritable (A: 0.3782 [0.3024, 0.4485]; E: 0.6218 [0.5515, 0.6976]). We confirmed the same directional effects for other diversity metrics for hippurate and the diet score (Supplementary Table S2).

### OTU and collapsed taxa associations with hippurate

**Figure 1:**
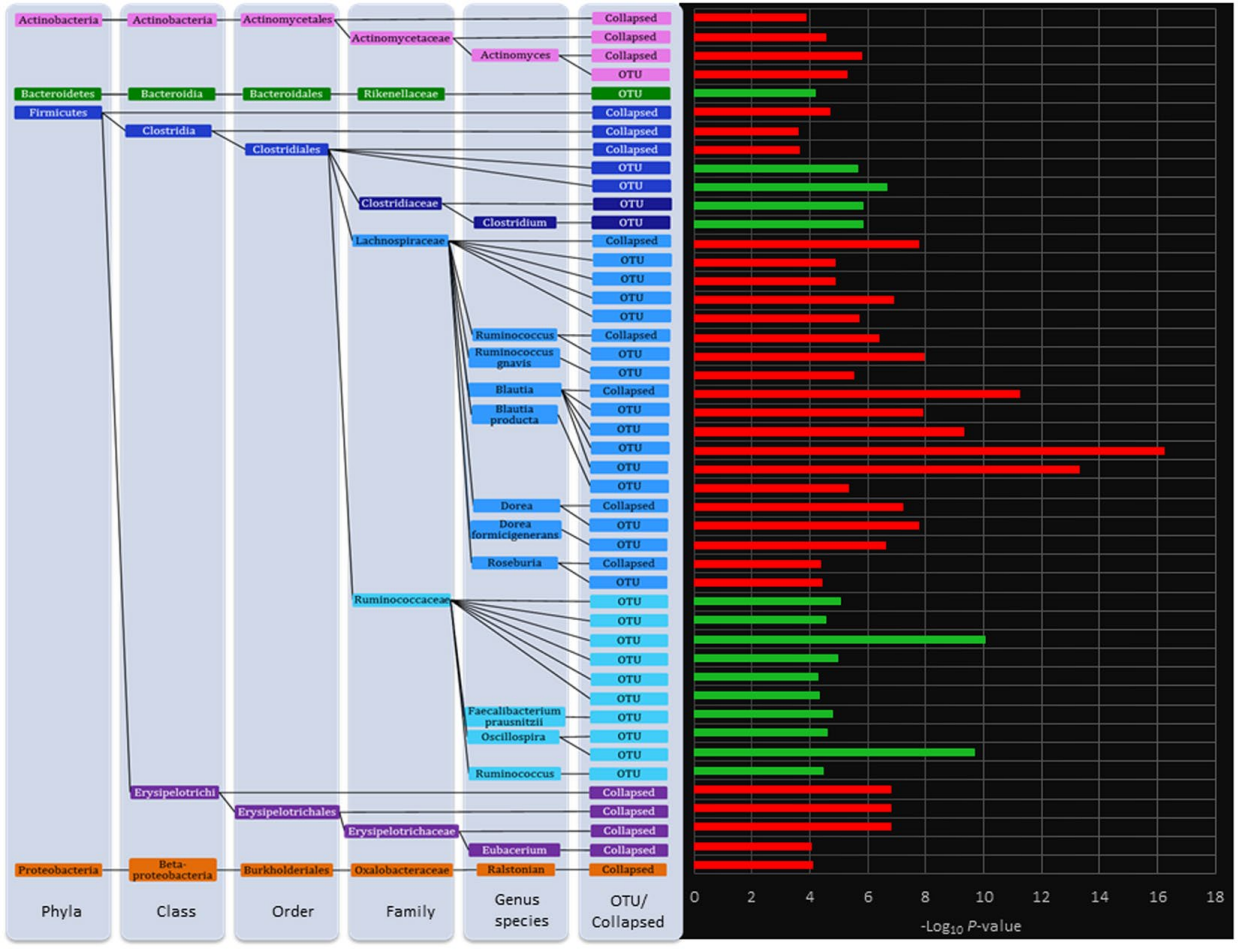
OTU and collapsed taxonomic associations with hippurate. Associations between blood hippurate and microbiome variables are represented the histogram bars on the right side of the plot. The histogram bars represent the −log_10_ of the *P*-value of the regression and the colour of the bars indicates the direction of association: green, positive; red, negative.

In the whole sample thirty OTUs and sixteen collapsed taxa (Fig. 1) were significantly associated with blood levels of hippurate (*P* < 8.61 × 10^−5^ [OTUs]−1.47 × 10^−3^ [phylum]). Direction of effect in significant OTU and taxa hits was consistent with taxonomic relationships across all results, except within Clostridia. However, this is a known polyphyletic taxon. The positively associated taxa or OTUs belonged to the Ruminococcaeae family and one OTU of the family Rikenellaceae. The negatively associated taxa or OTUs belonged to the class Erysipelotrichi, the order Actinomycetales, the Lachnospiraceae family and the collapsed *Ralstonian* genus.

Following a backward stepwise linear regression using *P* < 0.05 as the cut-off threshold, together the remaining OTUs accounted for 58.0% of the variance in Shannon diversity and 7.1% of the variance in hippurate levels (adjusted for diversity).

### OTU and Taxa associated with both hippurate and the hippurate diet score

Five OTUs and five taxa associated with hippurate were also associated with the diet score in the same direction (Table 2) in the whole sample. Specifically, reduced abundances of one OTU assigned to the genus *Actinomyces* were associated with an increased diet score, and similar to hippurate this trend was significant at both the family and order levels. At the genus level, reduced abundances of *Ruminococcus* (including 2 OTUs) and *Eubacterium* were associated with increasing diet scores. Moreover, increased diet scores were associated with increased abundances of OTUs assigned to the species *Faecalibacterium prausnitzii* and the genus *Clostridiales*. Most associations appeared to be to be primarily related to intakes of fruit and whole grains (Table 2). Though higher abundances of one Clostridiales OTU were associated with increased coffee intake.

**Table 2:** List of taxa associated with hippurate, the hippurate diet score and foods^1^.

Phylum	Class	Order	Family	Genus species	OTU/Collapsed^2^	Hippurate		Diet score		Foods^3^
						Beta (SE)	*P*	Beta (SE)	*P*	*P* < 0.05
Actinobacteria	Actinobacteria	Actinomycetales			Collapsed	−0.083(0.022)	1.31 × 10^−4^	−0.035(0.011)	1.67 × 10^−3^	Fruit: −0.004(0.002) WG: −0.007(0.003)
Actinobacteria	Actinobacteria	Actinomycetales	Actinomycetaceae		Collapsed	−0.089(0.021)	2.89 × 10^−5^	−0.036(0.011)	1.70 × 10^−3^	Fruit: −0.004(0.002) WG: −0.007(0.003)
Actinobacteria	Actinobacteria	Actinomycetales	Actinomycetaceae	*Actinomyces*	Collapsed	−0.101(0.021)	1.55 × 10^−6^	−0.045(0.011)	5.71 × 10^−5^	Fruit: −0.005(0.002) WG: −0.008(0.003)
Actinobacteria	Actinobacteria	Actinomycetales	Actinomycetaceae	*Actinomyces*	OTU	−0.099(0.022)	5.14 × 10^−6^	−0.051(0.011)	2.81 × 10^−6^	Fruit: −0.005(0.002) WG: −0.009(0.003)
Firmicutes	Clostridia	Clostridiales			OTU	0.113(0.024)	2.21 × 10^−6^	0.044(0.010)	9.76 × 10^−6^	Coffee: 0.013(0.002)*
Firmicutes	Clostridia	Clostridiales	Lachnospiraceae	*Ruminococcus*	Collapsed	−0.111(0.022)	4.03 × 10^−7^	−0.038(0.011)	6.35 × 10^−4^	Fruit: −0.005(0.002) WG: −0.008(0.003)
Firmicutes	Clostridia	Clostridiales	Lachnospiraceae	*Ruminococcus*	OTU	−0.123(0.021)	1.17 × 10^−8^	−0.054(0.011)	2.79 × 10^−6^	Fruit: −0.006(0.002)* WG: −0.009(0.003)
Firmicutes	Clostridia	Clostridiales	Lachnospiraceae	*Ruminococcus gnavis*	OTU	−0.107(0.023)	3.04 × 10^−6^	−0.064(0.011)	1.99 × 10^−8^	Fruit: −0.006(0.002)* WG: −0.009(0.003)
Firmicutes	Clostridia	Clostridiales	Ruminococcaceae	*Faecalibacterium prausnitzii*	OTU	0.100(0.023)	1.66 × 10^−5^	0.034(0.010)	9.24 × 10^−4^	WG: 0.007(0.003)
Firmicutes	Erysipelotrichi	Erysipelotrichales	Erysipelotrichaceae	*Eubacterium*	Collapsed	−0.083(0.021)	9.30 × 10^−5^	−0.040(0.012)	6.12 × 10^−4^	Fruit: −0.004(0.002) WG: −0.010(0.003)*

^*^Statistically significant: *P* < 0.0017; WG: whole grain products.

^1^Microbiome OTUs and collapsed taxa significantly associated with both hippurate and the hippurate diet score (quartile-ranked, scored and summed intakes of coffee, fruit and whole grains) are shown. Associations were adjusted for covariates (age, Shannon Index, metabolite batch, BMI, sex and family relatedness) and multiple testing using Bonferroni correction. Hippurate diet score associations were also adjusted for hippurate.

^2^OTU or collapsed taxonomy.

^3^All foods included in the hippurate diet score were fitted into a backwards stepwise linear regression using *P* < 0.05 as the cut-off threshold with each taxa associated to both hippurate and the diet score. Results displayed are the betas with standard errors of foods at least nominally associated (*P* < 0.05). Statistical significance was defined as *P* < 0.0017 (Bonferroni: 0.05/[10 taxa × 3 foods]).

#### Relationship of diversity, the hippurate trend and diet to MetS and its components

Supplementary Table 3 provides the clinical characteristics of the subsample of individuals studied. Longitudinal hippurate trajectories were significantly associated with Shannon diversity, independently of diet and covariates in a subsample of 1032 individuals (15.736[1.96]; *P* = 4.95 × 10^−15^), moreover the hippurate trend accounted for 6.5% of the variance in Shannon diversity.

**Figure 2:**
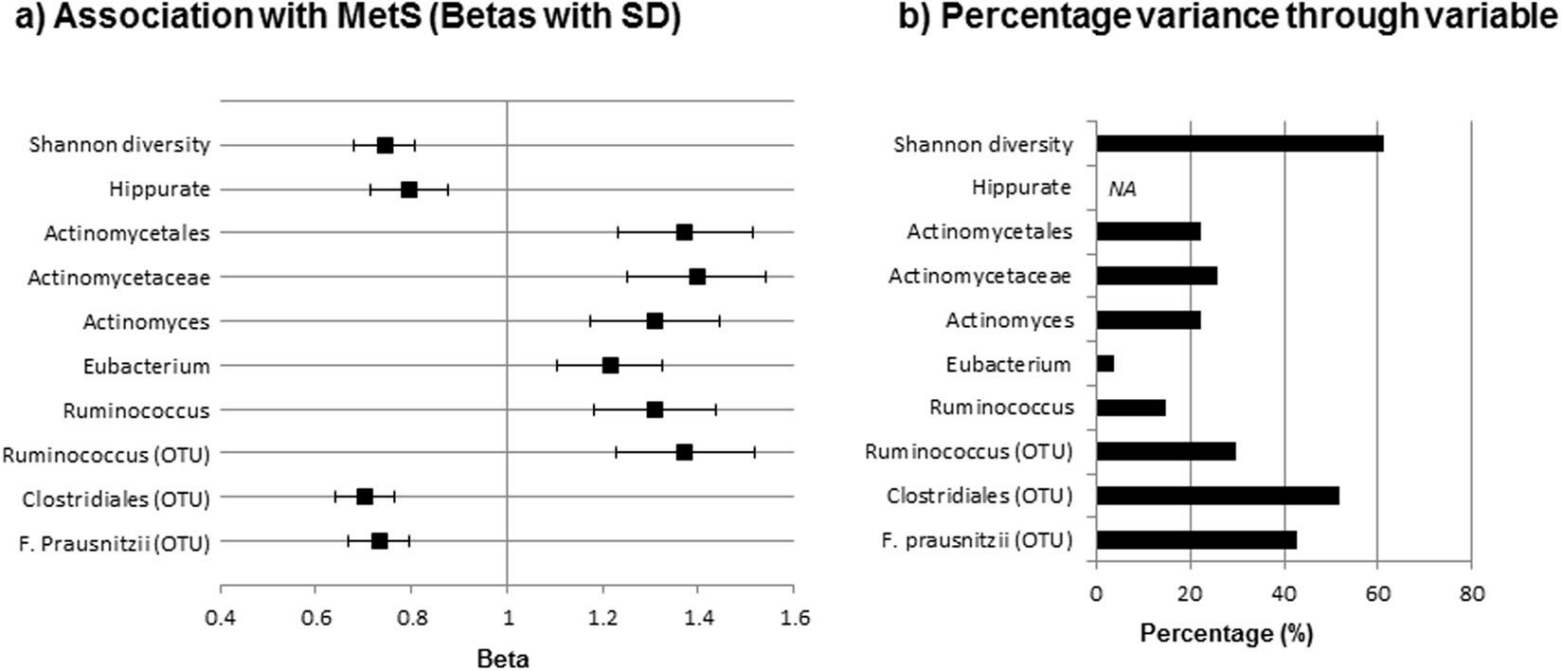
Associations between diversity, the hippurate trend, diet and OTUs and collapsed taxa with MetS status. (**a**) Shows the associations between MetS with Shannon diversity, the hippurate trend, and OTUs/taxa (significantly associated with hippurate, the diet score and MetS) represented as betas with SEs; all variables have been standardized. The diet score was not significantly associated with MetS. (**b**) Shows the percentage variance in the metabolite trend and MetS that was accounted for by the MetS association with Shannon diversity or associated OTUs/taxa. Abbreviations: MetS, metabolic syndrome; OTU, operational taxonomic unit; *NA*, not applicable.

Figure 2 shows the results of the analysis for associations between diversity, the hippurate trend and MetS. Higher Shannon diversity and an increasing hippurate trend were associated with a reduced risk of having MetS (Fig. 2a). 61.1% of the effect of the hippurate trend on MetS was accounted for by the association between Shannon diversity and MetS. Five collapsed taxa and 3 of the OTUs that were associated with both diet and hippurate were associated with MetS, including: the Actinomycetaceae family, and Actinomycetales order and *Actinomyces* genus within Actinomycetaceae, the *Eubacterium* and *Ruminococcus* genera (plus one OTU), which were positively associated; and OTUs assigned to the order Clostridiales and *Faecalibacterium prausnitzii* that were inversely associated. The percentage variance in the metabolite trend and MetS that was accounted for by the MetS association with these associated OTUs/taxa is shown in Fig. 2b.

#### Confirmation of results in discordant twins

We identified 55 MZ twin pairs who were discordant (1 SD apart) for Shannon diversity. Associations between Shannon diversity and hippurate were significant (*P* < 0.05) cross-sectionally (0.208[0.081]; *P* = 0.013) and longitudinally (0.478[0.078]; *P* = 9.53 × 10^−8^), and associations for top variables were in the same direction as in the much larger whole group analysis (Supplementary Table S4), except for HDL-cholesterol.

## Discussion

Here we have identified hippurate, through its relationship with microbiome diversity, as a potential modulator of MetS, independently of diet. Specifically, we find that circulating levels of five blood metabolites, including hippurate, are associated with greater gut microbiome diversity both cross-sectionally and longitudinally and higher intakes of fruit and whole grains. Hippurate and intakes of foods including coffee, fruit and whole grains also correlated with 5 OTUs and 5 collapsed taxa independent of diversity. In particular, hippurate was positively associated with an OTU within the order Clostridiales and the species *Faecalibacterium prausnitzii* and negatively with the Actinomycetaceae family, and the genera *Eubacterium* and *Ruminococcus*. Both an increasing Shannon diversity score and increasing hippurate levels were associated with a lower risk of MetS.

We identified and validated in an independent sample 5 metabolites associated with gut microbiome diversity: hippurate, p-cresol sulfate, phenylacetylglutamine and 3-phenylpropionate which were positively associated; and a negative association with hyodeoxycholate.

P-cresol sulfate (also p-cresyl sulfate) and phenylacetylglutamine are potentially toxic uremic solutes formed from the putrefication of undigested dietary proteins by colonic bacteria (and, in the case of p-cresol sulfate, subsequent modification by the liver). Our group previously showed higher p-cresol sulfate and phenylacetylglutamine levels to be strongly associated with early kidney dysfunction and gut microbiome OTUs^12^. Hyodeoxycholate is a secondary bile acid, produced from intestinal bacterial metabolism. Hyodeoxycholate is metabolised by glucuronidation in the human liver and kidneys, a pathway for toxin elimination^13^. Both 3-phenylpropionate and hippurate are derived from gut microbial metabolism of polyphenols to benzoates, though hippurate accounted for a larger portion of the variance in diversity. Hippurate is a glycine conjugate of benzoic acid formed in the mitochondria of the liver and kidneys^14^, and also through gut bacterial metabolism of dietary components, primarily polyphenols^15,16^. Although hippurate is also derived from the metabolism of quinic acid and/or shikimic acid^17^.

Overall, increased Shannon diversity and the hippurate trend were associated with a reduced odds of having MetS. Interestingly, 61.1% of the effect of the hippurate trend on MetS was mediated by the association between Shannon diversity and MetS. A previous study has shown reduced gut bacterial diversity of MetS features^6^. We believe the relationship between MetS and blood hippurate to be a novel finding, though studies have shown reduced urinary hippuric acid excretion in obesity^18–20^. We have previously found increasing circulating levels of hippurate to be associated with adipose tissue gene expression levels of neuroglobin, a type of globin^21^. Neuroglobin expressed in neurons and some endocrine tissues acts to protect cells against hypoxia and oxidative stress^22^.

Overall increased abundances OTUs/taxa of the Actinomycetaceae family and the genera *Eubacterium* and *Ruminococcus* were associated with reduced hippurate and the diet and increased MetS risk, and increased abundances of OTUs of the order Clostridiales and the species *Faecalibacterium prausnitzii* were associated with increased hippurate and diet score and reduced MetS risk.

The Actinomycetaceae family are typical commensals present within the oral cavity. In rare cases, an *Actinomyces* overgrowth contributes to an infection within the gut through forming filamentous branches that grow through damaged mucosal tissue penetrating the gut barrier, forming abscesses and fistula^23^. The relationship between *Actinomycetaceae* and the foods forming the hippurate diet score is not entirely clear. We found increased abundance of the genus *Eubacterium* to be associated with lower hippurate and the diet score, particularly whole grain intake. Contrary to our findings, feeding of whole grains^24^ and switching from a Western to plant-based diet^25^ in 10 humans have enriched abundances of species *Eubacterium rectale*. Another species, *Eubacterium dolichum* was found to be elevated in mice fed a Western-style diet^26^. Higher abundances of the genus *Ruminococcus* (one OTU and collapsed taxonomy) were associated with higher MetS risk. Increased *Ruminococcus* abundances were associated with lower fruit intake. *Ruminococcus* abundances were reduced following 12-week feeding of *schisandra chinensis* fruit, which is high in flavonoids^27^.

Increased abundances of OTUs within Clostridiales and *Faecalibacterium prausnitzii* were associated with increased hippurate, dietary components and reduced risk of MetS. The Clostridiales OTU was strongly and positively associated with coffee intake. Significantly elevated levels of the *Clostridium coccoides*-*Eubacterium rectale* group have been shown following the incubation of human faecal microbiota with coffee samples^28^. *Faecalibacterium prausnitzii* has been shown to correlate with microbiome gene count and predict weight loss over time^6^. *Faecalibacterium prausnitzii* has been shown to be depleted in 239 MetS subjects and partially restored following a 2 year Mediterranean diet intervention^29^. Increased abundances of the *Faecalibacterium prausnitzii* OTU were mildly associated with higher whole grain intake. Whole grains appear to allow *Faecalibacterium prausnitzii* to flourish^30^.

There were a number of limitations to this study. Importantly, the metabolomics methods utilised in our study do not yield absolute concentrations. Ideally a targeted method that could provide robust validation of the quantitative results. These data however offer important insights that can be tested by other groups using targeted metabolomics methods. The use of targeted assays would have been an ideal way to validate our findings. Unfortunately it is not a viable option for our cohort. We included few males therefore these results may only apply to women. As the FFQ relies on subject reporting, the accuracy of this recall data is always to some extent problematic. If misclassification of intakes had occurred, it would appear as error and likely have obscured any real findings and not strengthened them. Furthermore, we replicated our findings in MZ twin pairs and from previous feeding studies which have shown hippurate excretion to be increased following the consumption of these foods. We have not considered the influence of freezing and transit time in these analyses. Whilst these may influence results we do not expect this to be a large effect as variations in sample collection would be distributed randomly in relation to the phenotypes assessed. However, future analyses could be improved by taking these into consideration in the experimental design and analysis stages. Moreover, it will be of interest to carry out further research to identify how the bacteria interact and how that might influence microbiome diversity, related metabolites and be affected by diet. Finally, the range of time differences between sample collections is an issue throughout this paper. Though likely our results would have been stronger if closer time points were used between data collections.

There were also many advantages. We had a large number of twin subjects with the unique combination of metabolomics profiling, dietary information and gut microbiome profiling. We also had access to a unique longitudinal metabolomics dataset in order to evaluate the influence of changes in hippurate levels on MetS risk.

In conclusion, we identified novel diet-microbiome-metabolite relationships including five specific metabolites that are related to a diverse microbiome profile. Hippurate in particular was strongly associated to increased gut microbiome diversity and consumption of polyphenol-rich foods including coffee, whole grains and fruit and reduced odds of MetS. The potential of hippurate as a marker of alpha-diversity and the interplay between a diet rich in these foods, gut microbes and hippurate production should now be established in dynamic mechanistic studies using a dietary intervention setting.

## Materials and Methods

**Figure 3:**
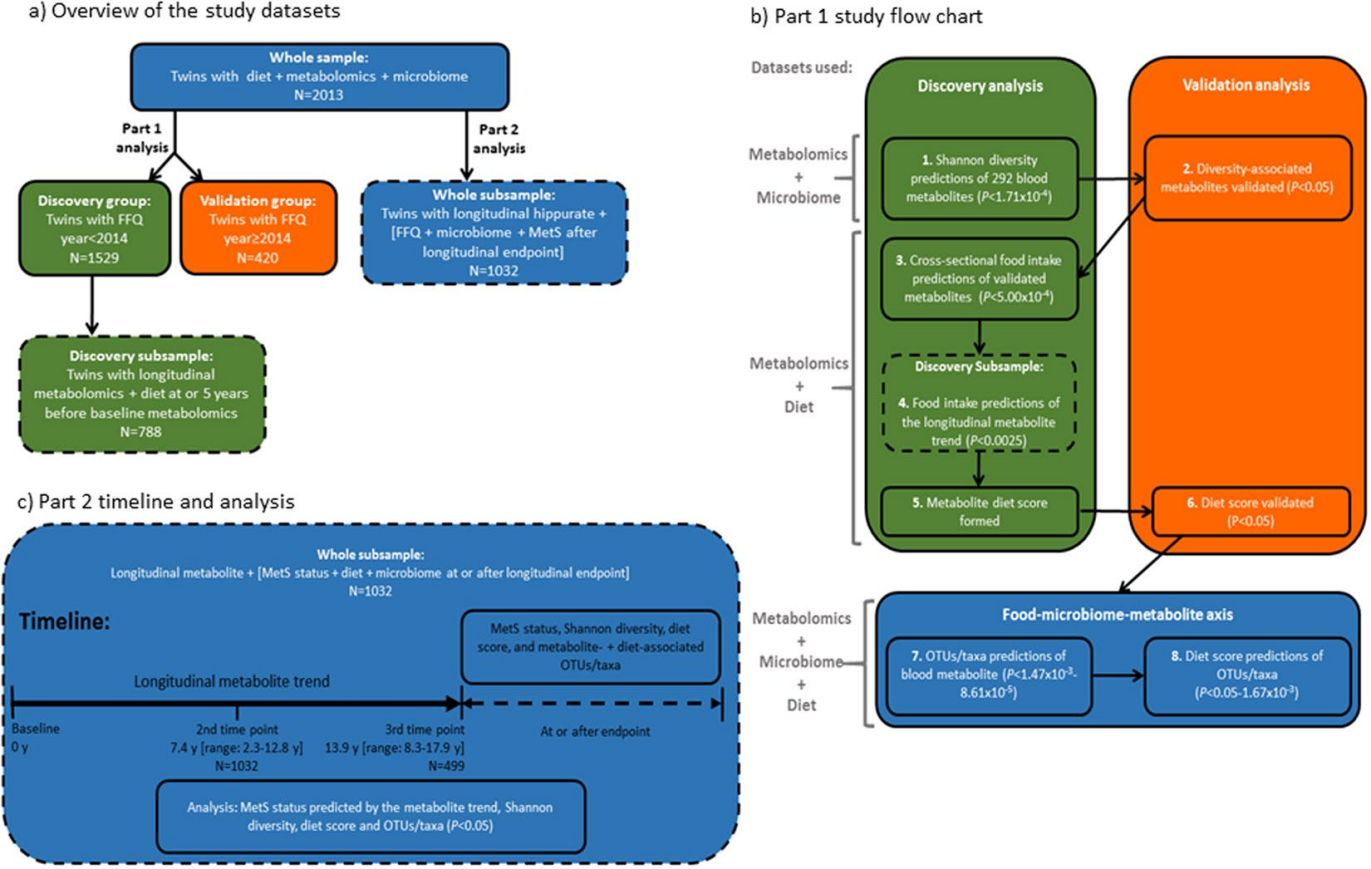
Overview of the study datasets and flow chart of study design. (**a**) Provides an overview of the study datasets. There were 5 different datasets used in the study. The colors and outline of the boxes indicate the datasets used; color: blue, whole; green, discovery; orange, validation; outline: solid, whole; dashed, subsample. All individuals included in the study had FFQ, blood metabolomics and microbiome data available. For part 1 cross-sectional analyses the whole sample was divided into discovery and validation groups based on when FFQs were completed. A subsample of individuals from the discovery group were used to examine baseline diet associations with longitudinal blood metabolomics. For part 2 analysis a subsample of individuals from the whole dataset were used to examine MetS associations with longitudinal metabolomics and cross-sectional diet and microbiome. (**b**) Shows the study outline for part 1 of the analysis where metabolite markers of microbiome diversity were identified and their relationship to diet examined. The flow chart is numbered in the order the analysis was conducted. On the left side of the figure the datasets used for each analysis step are indicated. (**c**) Shows the study outline for part 2 analysis where longitudinal levels of the top metabolite marker were examined for its relationship with MetS. Shannon diversity, the diet score, and metabolite- and diet-associated OTUs/taxa were investigated for their association with MetS status cross-sectionally.Abbreviations: MetS, metabolic syndrome; NA, not applicable.

The study population included twins enrolled in the TwinsUK registry, which is a national register of healthy adult twins residing throughout the UK^31^. Twins included in the dataset all had completed food intake questionnaires, faecal microbiome and blood metabolomics profiling (Fig. 3a shows the dataset information). Food intakes were estimated by administering a 131-item validated Food Frequency Questionnaire (FFQ)^32^ between 1995 and 2001, in 2007 and from 2014 to 2015. Food intake frequencies were collapsed into 20 different food types (Supplementary Table S5). The sample was divided into discovery and validation groups according to when FFQs were completed (before or in/after 2014). Female twins who completed FFQs collected between 1995 and 2001 and in 2007 were used in the discovery analysis (*n = *1529). New FFQ data was collected between 2014 and 2015; 484 additional individuals had microbiome and metabolomics data and completed FFQs during this time, 420 of these individuals who had no co-twin in the discovery sample were used as a validation sample. Quality control of the FFQ dataset has been outlined previously^33^. The study has been approved by the local St. Thomas’ Hospital Research Ethics Committee and was performed in accordance with the approved guidelines. All study participants provided written informed consent.

### Faecal microbiome composition

Faecal samples were collected at follow-up between 2012 and 2016, and the composition of the gut microbiome was determined by 16 S rRNA gene sequencing carried out as previously described^34^. Details for the microbiome quality control are described in Supplementary Text S1. Shannon diversity was the primary index considered for analysis, this index is normally distributed and most commonly used. There were on 5.46 (3.83) years between FFQ and faecal sample collection.

### Metabolomic profiling

Non-targeted mass spectroscopy-based metabolomic profiling was undertaken in three batches all within 2 years by the metabolomics provider Metabolon, Inc. (Durham, NC) on 6056 fasting blood samples (batch 1 in serum and batches 2 and 3 in plasma), as previously outlined^35^ and detailed in **Supplementary Text S2**. The Metabolon platform identified 292 structurally named biochemicals that belong to the following broad categories: amino acids, carbohydrates, vitamins, lipids, nucleotides, peptides, and xenobiotics (Supplementary Table S6). The associations between metabolite and batch adjusted for age, BMI and family relatedness can be found in Supplementary Table S7. Quality control on the metabolomics dataset was performed as previously described^35^ (Supplementary Text S2). Briefly, raw data were median-normalised by dividing the metabolite concentrations by the daily median for that metabolite. Metabolite concentrations did not follow a normal distribution and were therefore inverse-normalized. There were on average 3.13 (SD: 3.10) years between the FFQ and the blood sample collection.

### Classification of the metabolic syndrome

Clinical visits were undertaken where a trained research nurse or assistant collected blood samples, waist circumference and blood pressure (Supplementary Text S3 for details).

MetS status was determined by the International Diabetes Federation and the American Heart Association/National Heart, Lung, and Blood Institute criteria^36^.

### Data Availability

TwinsUK omics and phenotypic data are publicly available upon request. Details can be found on the departmental website (http://www.twinsuk.ac.uk/data-access/accessmanagement/).

### Statistical analysis

Statistical analysis was carried out using Stata version 12. The statistical analysis was undertaken in two parts. First, a metabolomic marker of microbiome diversity was identified, its relationship to food intake explored and associations with microbiome OTUs/collapsed taxonomies identified. In the second part, the relationship between longitudinal levels of the diversity metabolite marker, diet, diversity and associated OTUs/taxa with the risk of MetS was explored.

### Part 1: Metabolomics associations with diversity, relationship to food intake and associations with microbiome OTUs/taxa

Figure 3b shows the study outline for this section.

#### Microbiome diversity-metabolite associations

A linear mixed regression model with Shannon diversity as a predictor of the metabolite level was undertaken in a group of 1529 females, adjusted for age, batch, BMI and family relatedness as random intercept and multiple testing (Bonferroni: 0.05/292 = 1.71 × 10^−4^). Significant metabolites from the discovery sample were then evaluated against Shannon diversity using the same linear regression (additionally including sex as a covariate) in the validation sample (including n = 113 males), associations passing the 5% level of significance were considered validated.

#### Food intake associations with diversity-associated metabolites

In the discovery sample, reported intakes of 20 food groups were used to predict levels of the validated metabolites (Bonferroni: 0.05/[5 metabolites × 20 food groups] = 5.00 × 10^−4^), adjusted for the same covariates as above. In a subsample of individuals from the discovery group (*n* = 788) longitudinal metabolomics data was available (*n* = 705 3 time points, *n* = 83 2 time points) as well as reported food group intake at the same time or 5 years before the first blood sample. Trajectories of change (indicated as ‘trend’ from here forward) in the top diversity-associated metabolite (years after baseline: 2^nd^ time point: 7.4 [range: 2.3–12.8]; 3^rd^ time point: 13.9 [range: 8.3–17.9]) were determined by empirical Bayes predictions (adjusted for age and BMI) which estimates the rate of change in standard deviations/year^37^. Using this method, point estimates were calculated and a slope of change determined. Food group intake at or before baseline metabolite levels was then used to predict the metabolite trends (Bonferroni: 0.05/20 food groups = 0.0025).

A dietary score predictive of the metabolite trend was created from quartile (Q)-ranking significantly associated foods, scoring according to the direction of association (i.e. positive association: Q1 = 0-Q4 = 3; negative association: Q1 = 3-Q4 = 0) and summing. The heritability of the score was determined by structural equation modelling using Mx (Supplementary Text S4 for details).

To establish the association between the metabolite and diet score with richness (defined here as number of observed OTUs) and additional diversity metrics (Simpson and Chao1), these associations were run using the same linear regression as for the Shannon diversity discovery analysis, though in the whole sample.

#### Food-microbiome-metabolite axis

To identify associations between the metabolite and the microbiome, the discovery and validation samples were combined (*n* = 2013). Associations with the microbiome were evaluated by calculating a linear regression model using the OTUs and OTUs collapsed at each taxonomic level (phylum, class, order, family, genus) as predictors of the metabolite adjusted for covariates (age, BMI, batch, sex and family relatedness), Shannon diversity and multiple-testing (Bonferroni cut-off; Supplementary Table S8 shows the significance threshold for the OTUs and each taxonomic level). To determine the total variance of both Shannon diversity and the metabolite explained by the metabolite-associated OTUs, we included all associated OTUs in a backwards stepwise linear regression using *P* < 0.05 as the threshold cut-off and we report theR^2^ for each model. We then calculated linear mixed models of metabolite-associated OTUs/taxa using the diet score as a predictor of abundances adjusted for covariates, the metabolite and multiple testing (assigned at each taxonomic level; Supplementary Table S8). To investigate if any of the foods forming the score were driving associations, we ran a multivariate regression model including all metabolite-associated foods and the same covariates.

### Part 2: The relationship between longitudinal levels of the diversity metabolite marker, diet, diversity and associated OTUs/taxa with the risk of MetS

Figure 3c shows the analysis pipeline for this section.

#### Relationship between microbiome diversity, longitudinal metabolite and the MetS

A subsample of 1032 individuals (primarily female) had longitudinal levels of the blood metabolite (n = 533 with 2 time points, n = 499 with 3 time points; range: 2.4–17.9 years). To evaluate whether longitudinal levels of the top diversity metabolite predicted MetS status, we ran independent linear regression models each for Shannon diversity, the metabolite trend (determined using empirical Bayes predictions as above), the diet score, and metabolite/diet-associated OTUs/taxa to predict MetS status adjusting for age, sex, and family relatedness.

#### Longitudinal Metabolite association with MetS mediated by Shannon diversity, the diet score and specific taxa

The aim of this analysis was to determine how much of the variance in the metabolite trend and MetS was accounted for by the MetS association with Shannon diversity, the diet score or specific OTUs/taxa. Details are provided in **Supplementary Text S5**.

#### Confirmation of findings in twins discordant for Shannon diversity

In 55 MZ twin pairs discordant (≥1 SD) for Shannon diversity, we confirmed associations between Shannon diversity and the longitudinal metabolite trend, also cross-sectional metabolite, diet score and MetS using the same regression models as the discovery analysis.

## Electronic supplementary material

Supplementary Information
