# Supplementary material for: Hippurate as a metabolomic marker of gut microbiome diversity: Modulation by diet and relationship to metabolic syndrome
Source: Sci Rep. 2017 Oct 20;7:13670. doi: 10.1038/s41598-017-13722-4 (PMC5651863; doi:10.1038/s41598-017-13722-4)
Supplement: Supplementary file 1 — Supplementary Information [file 41598_2017_13722_MOESM1_ESM.doc]

**Supplementary Text and Tables**

**Hippurate as a metabolomic marker of gut microbiome diversity: Modulation by diet and relationship to metabolic syndrome**

Tess Pallister, Matthew A Jackson, Tiphaine C Martin, Jonas Zierer, Amy Jennings, Robert P Mohney, Alexander MacGregor, Claire J Steves, Aedin Cassidy, Tim D Spector, Cristina Menni

**Supplementary Table S1**. Study population characteristics for the whole, discovery and validation samples

|  | Whole |  | FFQ<2014  (Discovery) |  | FFQ≥2014  (including validation) |
| --- | --- | --- | --- | --- | --- |
|  | Mean (SD) |  | Mean (SD) |  | Mean (SD) |
| N | 2013 |  | 1529 |  | 484 |
| Age (y) | 57.2 (10.6) |  | 57.7 (10.6) |  | 55.4 (10.4) |
| BMI | 26.0 (4.6) |  | 26.1 (4.6) |  | 25.9 (4.6) |
| Sex (M:F) | 113:1900 |  | 0:1529 |  | 113:371 |
| MZ:DZ pairs:Singletons | 406:390  421 |  |  |  |  |
| Food groups (servings/week) |  |  |  |  |  |
| Vegetables | 34.9 (16.5) |  | 34.8 (15.4) |  | 35.1 (19.6) |
| Fruit | 21.4 (12.7) |  | 21.9 (12.4) |  | 19.5 (13.5) |
| Wholegrains | 10.3 (8.0) |  | 10.7 (8.1) |  | 9.2 (7.4) |
| Refined grains | 8.6 (7.6) |  | 9.0 (7.9) |  | 7.2 (6.3) |
| Nuts and legumes | 7.9 (5.8) |  | 7.6 (5.2) |  | 8.8 (7.2) |
| Seafood | 2.4 (2.0) |  | 2.5 (2.0) |  | 2.3 (2.1) |
| White meat | 1.9 (1.3) |  | 1.9 (1.3) |  | 1.9 (1.3) |
| Red meat | 6.9 (4.1) |  | 6.8 (3.9) |  | 7.4 (4.6) |
| Fermented dairy | 6.2 (5.1) |  | 6.1 (4.7) |  | 6.5 (6.2) |
| Fried and fast foods | 4.5 (3.5) |  | 4.5 (3.3) |  | 4.8 (4.1) |
| Sweets and sweet baked products | 15.2 (13.9) |  | 15.5 (14.0) |  | 14.1 (13.4) |
| Chocolate | 4.0 (5.9) |  | 4.0 (5.8) |  | 3.9 (6.3) |
| Butter and cream | 4.4 (6.5) |  | 4.2 (6.4) |  | 5.0 (6.8) |
| Spreads and dressings | 8.2 (9.0) |  | 8.5 (9.1) |  | 7.2 (8.6) |
| Milk | 3.2 (2.3) |  | 3.4 (2.3) |  | 2.8 (2.2) |
| Soya and other milk | 0.2 (0.8) |  | 0.2 (0.9) |  | 0.2 (0.8) |
| Soda | 1.7 (4.2) |  | 1.8 (4.3) |  | 1.3 (3.5) |
| Tea | 19.3 (13.5) |  | 19.2 (13.5) |  | 19.4 (13.5) |
| Coffee | 9.0 (10.6) |  | 9.1 (10.7) |  | 8.8 (10.5) |
| Alcohol | 6.1 (8.0) |  | 6.2 (7.9) |  | 5.9 (8.4) |

**Supplementary Table S2**. Associations between hippurate and the hippurate diet score across diversity metrics in the whole sample

|  | **Hippurate ^(2)^** | |  | **Hippurate diet score ^(3)^** | |
| --- | --- | --- | --- | --- | --- |
| **Diversity metric ^(1)^** | Beta(SE) | *P* |  | Beta(SE) | *P* |
| Observed species | 0.158 (0.024) | 9.55x10^-11^ |  | 0.086 (0.023) | 2.17x10^-4^ |
| Shannon | 0.160 (0.025) | 2.16x10^-10^ |  | 0.108 (0.023) | 3.03x10^-6^ |
| Simpson | 0.082 (0.026) | 0.002 |  | 0.062 (0.021) | 0.003 |
| Chao1 | 0.060 (0.023) | 0.011 |  | 0.023 (0.023) | *NS* |

*NS*= not significant: *P*>0.05.

1. Standardized to have mean 0, SD 1.
2. Hippurate associations with diversity metrics adjusted for sex, age, BMI, metabolite batch and family relatedness. Hippurate diet score associations with diversity adjusted for sex, hippurate, age, BMI, metabolite batch and family relatedness.

| **Supplementary Table S3. Clinical characteristics of the twin subsample (n=1032) to investigate**  **longitudinal hippurate, diversity, and diet with the MetS phenotypes and its components** | |
| --- | --- |
| Variable | Mean (SD) |
| Age at MetS status (y) | 64.2 (7.8) |
| MetS status (0, no; 1, yes) | 906:116 |
| Longitudinal metabolomics baseline to endpoint (y) | 10.6 (3.9) |
| Sex (M:F) | 27:1005 |
| BMI (kg/m2) | 26.2 (4.5) |
| Systolic blood pressure (mmHg) | 129.0 (15.7) |
| Diastolic blood pressure (mmHg) | 75.5 (9.7) |
| Glucose (mmol/L) | 4.9 (0.6) |
| Cholesterol (mmol/L) | 5.6 (1.0) |
| HDL-Cholesterol (mmol/L) | 1.9 (0.5) |
| Triglycerides (mmol/L) | 1.1 (0.5) |
| Type 2 Diabetes Mellitus (n) | 19 |
| Blood pressure medication (n) | 229 |
| Cholesterol medication (n) | 1 |

MetS, metabolic syndrome; HDL, high density lipoprotein

**Supplementary Table S4. Associations between diversity and hippurate (discovery), the hippurate trajectory, MetS status and components in MZ twins discordant for diversity**

| Variable ^(1)^ | Beta(SE) ^(2)^ | *P* | R^2^ |
| --- | --- | --- | --- |
| Hippurate (discovery) | 0.208 (0.081) | 0.013 | 0.0607 |
| Hippurate trajectory | 0.478 (0.078) | 9.53x10^-8^ | 0.1768 |
| Hippurate diet score | 0.149 (0.099) | 0.137 | 0.0136 |
| MetS status* | 1.046 (0.299) | 0.875 | 0.0004 |
| BMI | 0.021 (0.063) | 0.737 | 0.0010 |
| HDL-cholesterol | -0.035 (0.069) | 0.612 | 0.0022 |
| TG | -0.073 (0.060) | 0.233 | 0.0118 |

1. MetS, metabolic syndrome; HDL, high density lipoprotein; TG, triglycerides
2. A linear regression was conducted using Shannon diversity to predict hippurate (discovery), the hippurate trajectory, and MetS status and components in the MZ discordant (1 SD apart in diversity) twin sample.

*Statistical results show the odds ratio. Variables were standardized to have mean=0, SD=1

**Supplementary Table S5**. Food items included in food groups

| **Food group** | **FFQ items** |
| --- | --- |
| **Vegetables** | |
|  | Broccoli, spring green, kale |
|  | Brussel sprouts |
|  | Cabbage |
|  | Cauliflower |
|  | Coleslaw |
|  | Avocado |
|  | Beetroot |
|  | Marrow, courgettes |
|  | Mushrooms |
|  | Parsnips, turnips, swedes |
|  | Sweetcorn |
|  | Sweet peppers |
|  | Watercress |
|  | Carrots |
|  | Tomatoes |
|  | Garlic (clove) |
|  | Leeks |
|  | Onions |
|  | Green salad, lettuce, cucumber, celery |
|  | Spinach |
|  | Watercress |
|  | Vegetable soups (bowl) |
|  | Boiled, mashed, instant or one jacket potato |
| **Fruit** | |
|  | Strawberries, raspberries, other berries, kiwi fruit (one fruit or handful) |
|  | Smoothies (cup) |
|  | Pure fruit juice (100%) e.g. orange, apple juice (cup) |
|  | Grapefruit (half) |
|  | Oranges, satsumas, mandarins (1 fruit) |
|  | Apples (1 fruit) |
|  | Bananas (1fruit) |
|  | Dried fruit, e.g. raisins, prunes (heaped tablespoon) |
|  | Grapes (handful) |
|  | Melon (1 slice) |
|  | Peaches, plums, apricots (1 fruit) |
|  | Pears (1 fruit) |
|  | Tinned fruit (handful) |
| **Whole grains** | |
|  | High Fibre cereals e.g. Branflakes, All Bran, Fruit and Fibre |
|  | Muesli |
|  | Porridge, Readybreak, oats |
|  | Brown rice |
|  | Wholemeal & granary bread/rolls |
|  | Wholemeal pasta |
|  | Crispbread, e.g. Ryvita |
| **Refined grains** | |
|  | Breakfast cereal e.g. Cornflakes, Rice Krispies |
|  | Sugar topped cereals e.g. Frosties |
|  | Naan, poppadoms, flour tortillas |
|  | Brown bread/rolls |
|  | White bread/rolls |
|  | White or green pasta, e.g. spaghetti, macaroni, noodles |
|  | White rice |
| **Nuts and legumes** | |
|  | Beansprouts |
|  | Pulses e.g. lentils, beans, peas |
|  | Green beans, broad beans, runner beans |
|  | Peas |
|  | Baked beans |
|  | Salted nuts e.g. peanuts, cashews (handful) |
|  | Unsalted nuts, e.g. brazil, walnuts (handful) |
|  | Seeds e.g. Sunflower, pumpkin (tablespoon) |
|  | Peanut butter (teaspoon) |
|  | Meat substitutes e.g. tofu, soyameat, textured vegetable protein, vegeburger |
| **Seafood** | |
|  | Oily fish, fresh or canned, e.g. tuna, mackerel, kippers, salmon, sardines, herring |
|  | Fish roe, taramasalata |
|  | Shellfish, e.g. crab, prawns, mussels |
|  | Other white fish, fresh or frozen, e.g. cod, plaice, sole, haddock, halibut |
| **White meat** | |
|  | Chicken or other poultry e.g. turkey |
| **Red, processed meat and eggs** | |
|  | Beef: roast, steak, mince, stew or casserole |
|  | Lamb: roast, chops or stew |
|  | Pork: roast, chops or stew |
|  | Beefburgers |
|  | Bacon or gammon |
|  | Corned Beef, Spam, luncheon meats |
|  | Ham, cured meats & chorizo |
|  | Liver, liver pate, liver sausage |
|  | Sausages |
|  | Eggs as boiled, fried, scrambled, etc. (one) |
| **Fermented dairy** | |
|  | Low fat cheese e.g. reduced fat cheddar (matchbox size) |
|  | Cheese, e.g. cheddar, brie, edam (matchbox size) |
|  | Cottage cheese, low fat soft cheese (2 tablespoons) |
|  | Full fat or Greek yoghurt (small pot) |
|  | Low fat yoghurt, fromage frais (small pot) |
| **Fried and fast foods** | |
|  | Fish fingers, fish cakes & breaded fish |
|  | Fried fish in batter, as in fish and chips |
|  | Chips, retail, fried in vegetable oil |
|  | Potato salad |
|  | Old potatoes, roast in blended oil |
|  | Savoury pies, e.g. meat pie, pork pie, pasties, steak & kidney pie, sausage rolls |
|  | Cream crackers, savoury biscuits |
|  | Crisps or other packet snacks, e.g. Wotsits (one packet) |
|  | Pizza (one slice) |
|  | Quiche (slice) |
| **Sweets and sweet baked products** | |
|  | Reduced fat biscuits e.g. Go Ahead, Highlights (one small packet or one small bar/biscuit) |
|  | Sweet biscuits, chocolate, e.g. digestive (one) |
|  | Sweet biscuits, plain, e.g. Nice, ginger (one) |
|  | Buns, pastries e.g. scones, flapjacks, croissants, doughnuts, home baked |
|  | Cakes e.g. fruit, sponge, home baked |
|  | Cakes e.g. fruit, sponge, ready made |
|  | Fruit pies, tarts, crumbles, home baked |
|  | Fruit pies, tarts, crumbles, ready made |
|  | Milk puddings e.g. rice, custard, trifle |
|  | Sponge puddings, home baked |
|  | Sponge puddings, ready made |
|  | Dairy desserts (small pot) e.g. chocolate mousse, cream caramels |
|  | Ice cream, choc ices |
|  | Jam, marmalade, honey (teaspoon) |
|  | Sugar added to tea, coffee, cereal (teaspoon) |
|  | Sweets, toffees, mints (small packet) |
| **Chocolate** | |
|  | Dark chocolates, single or squares (one) |
|  | White or milk chocolates, single or squares (one) |
|  | Low fat hot chocolate (cup) |
|  | Cocoa, hot chocolate (cup) |
|  | Chocolate snack bars e.g. Mars, Crunchie (one) |
| **Butter and cream** | |
|  | Reduced fat butter (teaspoon) |
|  | Butter (teaspoon) |
|  | Double or clotted cream (tablespoon) |
|  | Single or sour cream (tablespoon) |
| **Spreads and dressings** | |
|  | Low fat spread, e.g. Outline, Gold (teaspoon) |
|  | Very low fat spread (teaspoon) e.g. Diet Flora |
|  | Cholesterol lowering fat spreads e.g. Benecol (teaspoon) |
|  | Olive oil spread (teaspoon) |
|  | Block margarine, e.g. Stork, Krona (teaspoon) |
|  | Other soft margarine, dairy spreads, e.g. Blue Band, Clover (teaspoon) |
|  | Polyunsaturated margarine, e.g. Flora, sunflower (teaspoon) |
|  | French dressing (tablespoon) |
|  | Full fat salad cream, mayonnaise (tablespoon) |
|  | Other salad dressing (tablespoon) |
|  | Low calorie, low fat salad cream (tablespoon) |
| **Milk** | |
|  | Channel Islands milk |
|  | Full cream milk |
|  | Dried milk |
|  | Semi-skimmed milk |
|  | Skimmed milk |
| **Soya and other milk** | |
|  | Goats' milk |
|  | Rice milk |
|  | Soya milk |
| **Soda** | |
|  | Fizzy soft drinks, e.g. Coca Cola, lemonade (cup) |
|  | Low calorie or diet fizzy soft drinks (cup) |
| **Tea** | |
|  | Tea (cup) |
|  | Green tea (cup) |
| **Coffee** | |
|  | Coffee, instant or ground (cup) |
|  | Coffee, decaffeinated (cup) |
| **Alcohol** | |
|  | Beer, lager or cider (half pint) |
|  | Port, sherry, vermouth, liqueurs (pub measure) |
|  | Spirits, e.g. gin, brandy, whisky, vodka (pub measure) |
|  | Red wine (small glass) |
|  | White wine (small glass) |

**Supplementary Text S1: Microbiome quality control procedures**

Briefly, the V4 region of the 16S rRNA gene was amplified and then sequenced on Illumina MiSeq. The reads were next compiled to OTUs (16). Quality control was undertaken by sample; paired-ends with an overlap of less than 200nt were removed using fastq join within QIIME. Chimeric sequences were then removed by de novo chimera detection in USEARCH (Edgar et al, 2011). Samples with less than 10,000 reads were discarded. Retained samples had a mean read depth of 81308 (sd = 38055). Using Sumaclust within QIIME 1.9.0 de novo OTU clustering was undertaken across all reads, reads with a 97% identity threshold were brought together (Jackson et al., 2016; Caporaso et al., 2010). Log transformation was undertaken on OTU relative abundances, adding a pseudocount to account for zero values (1x10^-6^). We discarded rare OTUs (found in less than 25% of samples) to focus on most the more common and more evenly distributed OTUs. OTU abundances were then adjusted for technical covariates including sequencing depth, sequencing run, sequencing technician and sample collection method using linear modelling and the residuals obtained. As the residuals were not normally distributed, an inverse normalisation was undertaken. Generation of collapsed taxonomies was carried out using all raw OTU counts with counts then transformed to abundances and adjusted as for OTUs. To determine alpha diversity, the total OTU count table was rarefied to 10000 sequences for each sample 50 times. Per sample, alpha diversity metrics were determined in each of the rarefied tables and the average score for all 50 was considered as the final diversity measure. Alpha diversity metrics considered were observed OTU counts, the Chao1 (richness index), the Shannon and Simpson indices. All alpha diversity indices were standardised to have mean 0 and SD 1. Shannon diversity was the primary index considered for analysis, this index is normally distributed and most commonly used.

References

Edgar RC, Haas BJ, Clemente JC, Quince C, Knight R. UCHIME improves sensitivity and speed of chimera detection. Bioinformatics (Oxford, England) 2011; 27:2194-2200

Jackson MA, Bell JT, Spector T, Steves C. A heritability-based comparison of methods used to cluster 16S rRNA gene sequences into operational taxonomic units. Peer J Preprints 2016;

Caporaso JG, Kuczynski J, Stombaugh J, Bittinger K, Bushman FD, Costello EK, Fierer N, Pena AG, Goodrich JK, Gordon JI, Huttley GA, Kelley ST, Knights D, Koenig JE, Ley RE, Lozupone CA, McDonald D, Muegge BD, Pirrung M, Reeder J, Sevinsky JR, Turnbaugh PJ, Walters WA, Widmann J, Yatsunenko T, Zaneveld J, Knight R. QIIME allows analysis of high-throughput community sequencing data. Nature methods 2010; 7:335-336

**Supplementary Text S2: Information for Metabolon metabolomics profiling**

**Sample preparation for global metabolomics profiling**

**N**on-targeted mass spectroscopy-based metabolomic profiling was undertaken by the metabolomics provider Metabolon, Inc. (Durham, NC) on 6056 fasting blood samples. Samples were stored at -70°C until processed. Recovery standards were added prior to the first step in the extraction process for quality control purposes. To remove protein, dissociate small molecules bound to protein, and recover chemically diverse metabolites, proteins were precipitated with methanol under vigorous shaking for 2 min (Glen Mills Genogrinder 2000) followed by centrifugation. The resulting extract was divided into four fractions: one for analysis by ultra high performance liquid chromatography-tandem mass spectrometry (UPLC-MS/MS; positive mode), one for analysis by UPLC-MS/MS (negative mode), one for analysis by gas chromatography-mass spectrometry (GC-MS), and one sample was reserved for backup.

Three types of controls were analyzed in concert with the experimental samples: samples generated from a pool of human plasma extensively characterized by Metabolon, Inc. served as technical replicates throughout the data set; extracted water samples served as process blanks; and a cocktail of standards spiked into every sample allowed for instrument performance monitoring. Experimental samples and controls were randomized across the platform run.

**Mass spectrometry analysis**

Extracts were subjected to either GC-MS or UPLC-MS/MS using standardized chromatography. Vacuum-dried samples were dissolved in injection solvent containing eight or more injection standards at fixed concentrations, depending on the platform, to assure injection and chromatographic consistency. Instruments were tuned and calibrated for mass resolution and mass accuracy daily.

The UPLC-MS/MS platform utilized a Waters Acquity UPLC and a ThermoFisher LTQ mass spectrometer, which included an electrospray ionization source (ESI) and a linear ion-trap mass analyzer operated at nominal mass resolution. The instrumentation was set to monitor for positive ions in acidic extracts or negative ions in basic extracts through independent injections. Extracts were reconstituted, loaded onto columns heated to 40 °C (Waters UPLC BEH C18-2.1×100 mm, 1.7 µm), and gradient-eluted with water and 95% methanol containing 0.1% formic acid (acidic extracts) or 6.5 mM ammonium bicarbonate (basic extracts), as outlined previously (Evans, A.M. et al., 2009). Briefly, the extracts that were reconstituted in formic acid were gradient-eluted at 350 μL/min using (A) 0.1% formic acid in water and (B) 0.1% formic acid in methanol (0% B to 70% B in 4 min, 70-98% B in 0.5 min, 98% B for 0.9 min), whereas the extracts reconstituted in ammonium bicarbonate used (A) 6.5 mM ammonium bicarbonate in water, pH 8, and (B) 6.5 mM ammonium bicarbonate in 95/5 methanol/water (same gradient profile as above) also at 350 μL/min. A 5 μL aliquot of sample was injected using 2× overfill. Columns were washed and reconditioned after every injection.  The MS interface capillary was maintained at 350 °C, with a sheath gas flow of 40 (arbitrary units) and auxiliary gas flow of 5 (arbitrary units) for both positive and negative injections. The spray voltage for the positive ion injection was 4.5 kV, and it was 3.75 kV for the negative ion injection. The instrument was set to scan 99–1000 m/z and alternated between MS and data-dependent MS/MS scans using dynamic exclusion. The scan speed was approximately six scans per second (three MS and three MS/MS scans). The MS scan had an ion-trap target of 2 × 10^4^ (arbitrary units) and an ion-trap fill time cutoff of 200 ms. The MS/MS scan had an ion-trap target of 1 × 10^4^ (arbitrary units) and an ion-trap fill time cutoff of 100 ms. MS/MS normalized collision energy was set to 40, activation Q 0.25, and activation time 30 ms, with a 3 m/z isolation window.

The samples destined for analysis by GC-MS were dried under vacuum desiccation for a minimum of 18 h prior to being derivatized under nitrogen using bistrimethyl-silyltrifluoroacetamide. Derivatized samples were separated on a 5% phenyldimethyl silicone column (20m x 0.18mm x 0.18um df) with helium as the carrier gas (flow rate of 0.6 ml/min) and a linear temperature ramp from 60°C to 340°C within a 17-min period (i.e., 60°C, hold for 1.0 min, ramp to 220°C at a rate of 17.1°C per min, then ramp to 340°C at a rate of 30°C per min, hold for 3.67 min) using a split injection. All samples were analyzed on a Thermo-Finnigan Trace DSQ MS operated at unit mass resolving power with electron impact ionization and a 50–750 atomic mass unit scan range.

**Compound identification and quantification**

Metabolites were identified by automated comparison of the ion features in the experimental samples to a reference library of >4000 purified chemical standard entries that included retention time, molecular weight (m/z), preferred adducts, and in-source fragments as well as associated MS spectra. All metabolites reported in this study conform to the confidence Level 1 (the highest confidence level of identification) of the Metabolomics Standards Initiative (Sumner et al., 2007 and Schrimpe-Rutledge et al., 2016). Metabolomics data were curated by visual inspection for quality control using software developed at Metabolon (DeHaen et al., 2010). The Metabolon platform identified 292 structurally named biochemicals that belong to the following broad categories: amino acids, carbohydrates, vitamins, lipids, nucleotides, peptides, and xenobiotics.

Peaks were quantified using area-under-the-curve. Raw area counts for each metabolite in each sample were normalized to correct for variation resulting from instrument inter-day tuning differences by the median value for each run-day, therefore, setting the medians to 1.0 for each run. This preserved variation between samples but allowed metabolites of widely different raw peak areas to be compared on a similar graphical scale. Missing values were imputed with the observed minimum after normalization. Metabolite concentrations did not follow a normal distribution and were therefore inverse-normalized (Menni et al., 2013).

References

Evans, A.M. et al., Integrated, nontargeted ultrahigh performance liquid chromatography/electrospray ionization tandem mass spectrometry platform for the identification and relative quantification of the small-molecule complement of biological systems. *Analytical Chemistry* 81, 6656-6667, doi: 10.1021/ac901536h (2009).

Sumner, L. W. *et al*. Proposed minimum reporting standards for chemical analysis. *Metabolomics* 3, 211-221, doi: 10.1007/s11306-007-0082-2 (2007).

Schrimpe-Rutledge, A. C., Codreanu, S. G., Sherrod, S. D. & McLean, J. A. Untargeted Metabolomics Strategies – Challenges and Emerging Directions. *Journal of the American Society for Mass Spectrometry* 27, 1897-1905, doi: 10.1007/s13361-016-1469-y (2016)

DeHaven C. D., Evans A. M., Dai H, & Lawton K. A. Organization of GC/MS and LC/MS metabolomics data into chemical libraries. *Journal of Cheminformatics* 2, 9, doi: 10.1186/1758-2946-2-9 (2010).

Menni, C. et al. Metabolomic markers reveal novel pathways of ageing and early development in human populations. International journal of epidemiology 42, 1111-1119, doi:10.1093/ije/dyt094 (2013).

**Supplementary Table S6: Metabolites Analysed from the Metabolon Platform**

| **Metabolite name** | **Superpathway** | **Subpathway** |
| --- | --- | --- |
| **glutamine** | Amino acid | Glutamate metabolism |
| **tryptophan** | Amino acid | Tryptophan metabolism |
| **histidine** | Amino acid | Histidine metabolism |
| **leucine** | Amino acid | Valine, leucine and isoleucine metabolism |
| **cholesterol** | Lipid | Sterol, Steroid |
| **phenylalanine** | Amino acid | Phenylalanine & tyrosine metabolism |
| **creatinine** | Amino acid | Creatine metabolism |
| **lactate** | Carbohydrate | Glycolysis, gluconeogenesis, pyruvate metabolism |
| **3-hydroxybutyrate (BHBA)** | Lipid | Ketone bodies |
| **cotinine** | Xenobiotics | Tobacco metabolite |
| **caffeine** | Xenobiotics | Xanthine metabolism |
| **arabinose** | Carbohydrate | Nucleotide sugars, pentose metabolism |
| **fructose** | Carbohydrate | Fructose, mannose, galactose, starch, and sucrose metabolism |
| **mannose** | Carbohydrate | Fructose, mannose, galactose, starch, and sucrose metabolism |
| **pyruvate** | Carbohydrate | Glycolysis, gluconeogenesis, pyruvate metabolism |
| **uridine** | Nucleotide | Pyrimidine metabolism, uracil containing |
| **linoleate (18:2n6)** | Lipid | Essential fatty acid |
| **allantoin** | Nucleotide | Purine metabolism, urate metabolism |
| **arachidonate (20:4n6)** | Lipid | Long chain fatty acid |
| **deoxycholate** | Lipid | Bile acid metabolism |
| **margarate (17:0)** | Lipid | Long chain fatty acid |
| **isoleucine** | Amino acid | Valine, leucine and isoleucine metabolism |
| **threonine** | Amino acid | Glycine, serine and threonine metabolism |
| **tyrosine** | Amino acid | Phenylalanine & tyrosine metabolism |
| **lysine** | Amino acid | Lysine metabolism |
| **methionine** | Amino acid | Cysteine, methionine, SAM, taurine metabolism |
| **malate** | Energy | Krebs cycle |
| **palmitate (16:0)** | Lipid | Long chain fatty acid |
| **nonadecanoate (19:0)** | Lipid | Long chain fatty acid |
| **stearate (18:0)** | Lipid | Long chain fatty acid |
| **oleate (18:1n9)** | Lipid | Long chain fatty acid |
| **pentadecanoate (15:0)** | Lipid | Long chain fatty acid |
| **myristate (14:0)** | Lipid | Long chain fatty acid |
| **pipecolate** | Amino acid | Lysine metabolism |
| **ornithine** | Amino acid | Urea cycle |
| **5-oxoproline** | Amino acid | Glutathione metabolism |
| **pantothenate** | Cofactors and vitamins | Pantothenate and CoA metabolism |
| **salicylate** | Xenobiotics | Drug |
| **4-acetamidobutanoate** | Amino acid | Guanidino and acetamido metabolism |
| **alpha-tocopherol** | Cofactors and vitamins | Tocopherol metabolism |
| **citrate** | Energy | Krebs cycle |
| **glycerate** | Carbohydrate | Glycolysis, gluconeogenesis, pyruvate metabolism |
| **N-acetylalanine** | Amino acid | Alanine and aspartate metabolism |
| **urate** | Nucleotide | Purine metabolism, urate metabolism |
| **ursodeoxycholate** | Lipid | Bile acid metabolism |
| **arginine** | Amino acid | Urea cycle |
| **heptanoate (7:0)** | Lipid | Medium chain fatty acid |
| **laurate (12:0)** | Lipid | Medium chain fatty acid |
| **valine** | Amino acid | Valine, leucine and isoleucine metabolism |
| **urea** | Amino acid | Urea cycle |
| **cortisol** | Lipid | Sterol, Steroid |
| **cortisone** | Lipid | Sterol, Steroid |
| **proline** | Amino acid | Urea cycle |
| **citrulline** | Amino acid | Urea cycle |
| **biliverdin** | Cofactors and vitamins | Hemoglobin and porphyrin metabolism |
| **serotonin (5HT)** | Amino acid | Tryptophan metabolism |
| **gamma-glutamylglutamine** | Peptide | gamma-glutamyl |
| **gamma-glutamyltyrosine** | Peptide | gamma-glutamyl |
| **hypoxanthine** | Nucleotide | Purine metabolism, (hypo)xanthine, inosine containing |
| **betaine** | Amino acid | Glycine, serine and threonine metabolism |
| **xanthine** | Nucleotide | Purine metabolism, (hypo)xanthine, inosine containing |
| **caprate (10:0)** | Lipid | Medium chain fatty acid |
| **phosphate** | Energy | Oxidative phosphorylation |
| **3-methoxytyrosine** | Amino acid | Phenylalanine & tyrosine metabolism |
| **4-acetamidophenol** | Xenobiotics | Drug |
| **pelargonate (9:0)** | Lipid | Medium chain fatty acid |
| **undecanoate (11:0)** | Lipid | Medium chain fatty acid |
| **naproxen** | Xenobiotics | Drug |
| **beta-hydroxyisovalerate** | Amino acid | Valine, leucine and isoleucine metabolism |
| **glycerol** | Lipid | Glycerolipid metabolism |
| **kynurenine** | Amino acid | Tryptophan metabolism |
| **mannitol** | Carbohydrate | Fructose, mannose, galactose, starch, and sucrose metabolism |
| **glycerol 3-phosphate (G3P)** | Lipid | Glycerolipid metabolism |
| **acetylphosphate** | Energy | Oxidative phosphorylation |
| **carnitine** | Lipid | Carnitine metabolism |
| **choline** | Lipid | Glycerolipid metabolism |
| **N-acetylornithine** | Amino acid | Urea cycle |
| **N1-methyladenosine** | Nucleotide | Purine metabolism, adenine containing |
| **3-methyl-2-oxovalerate** | Amino acid | Valine, leucine and isoleucine metabolism |
| **3-methylhistidine** | Amino acid | Histidine metabolism |
| **3-phenylpropionate (hydrocinnamate)** | Amino acid | Phenylalanine & tyrosine metabolism |
| **hippurate** | Xenobiotics | Benzoate metabolism |
| **benzoate** | Xenobiotics | Benzoate metabolism |
| **phenylacetate** | Amino acid | Phenylalanine & tyrosine metabolism |
| **glycerophosphorylcholine (GPC)** | Lipid | Glycerolipid metabolism |
| **aspartate** | Amino acid | Alanine and aspartate metabolism |
| **X-04499--3,4-dihydroxybutyrate** | Amino acid | Butanoate metabolism |
| **ibuprofen** | Xenobiotics | Drug |
| **dihomo-linoleate (20:2n6)** | Lipid | Long chain fatty acid |
| **2-hydroxystearate** | Lipid | Fatty acid, monohydroxy |
| **paraxanthine** | Xenobiotics | Xanthine metabolism |
| **2-hydroxyhippurate (salicylurate)** | Xenobiotics | Benzoate metabolism |
| **quinate** | Xenobiotics | Food component, Plant |
| **indolelactate** | Amino acid | Tryptophan metabolism |
| **gamma-glutamylleucine** | Peptide | gamma-glutamyl |
| **theobromine** | Xenobiotics | Xanthine metabolism |
| **theophylline** | Xenobiotics | Xanthine metabolism |
| **eicosapentaenoate (EPA** | Lipid | Essential fatty acid |
| **estrone 3-sulfate** | Lipid | Sterol, Steroid |
| **glycocholate** | Lipid | Bile acid metabolism |
| **taurochenodeoxycholate** | Lipid | Bile acid metabolism |
| **taurocholate** | Lipid | Bile acid metabolism |
| **docosahexaenoate (DHA** | Lipid | Essential fatty acid |
| **1-stearoylglycerophosphoinositol** | Lipid | Lysolipid |
| **myo-inositol** | Lipid | Inositol metabolism |
| **glucose** | Carbohydrate | Glycolysis, gluconeogenesis, pyruvate metabolism |
| **1,5-anhydroglucitol (1,5-AG)** | Carbohydrate | Glycolysis, gluconeogenesis, pyruvate metabolism |
| **erythritol** | Xenobiotics | Sugar, sugar substitute, starch |
| **2-hydroxybutyrate (AHB)** | Amino acid | Cysteine, methionine, SAM, taurine metabolism |
| **3-methyl-2-oxobutyrate** | Amino acid | Valine, leucine and isoleucine metabolism |
| **1,6-anhydroglucose** | Carbohydrate | Glycolysis, gluconeogenesis, pyruvate metabolism |
| **1-palmitoylglycerol (1-monopalmitin)** | Lipid | Monoacylglycerol |
| **saccharin** | Xenobiotics | Food component, Plant |
| **1-oleoylglycerol (1-monoolein)** | Lipid | Monoacylglycerol |
| **1-stearoylglycerol (1-monostearin)** | Lipid | Monoacylglycerol |
| **2-hydroxyisobutyrate** | Amino acid | Valine, leucine and isoleucine metabolism |
| **4-methyl-2-oxopentanoate** | Amino acid | Valine, leucine and isoleucine metabolism |
| **phenyllactate (PLA)** | Amino acid | Phenylalanine & tyrosine metabolism |
| **homocitrulline** | Amino acid | Urea cycle |
| **aspartylphenylalanine** | Peptide | Dipeptide |
| **levulinate (4-oxovalerate)** | Amino acid | Valine, leucine and isoleucine metabolism |
| **palmitoylcarnitine** | Lipid | Carnitine metabolism |
| **cholate** | Lipid | Bile acid metabolism |
| **indoleacetate** | Amino acid | Tryptophan metabolism |
| **hyodeoxycholate** | Lipid | Bile acid metabolism |
| **3-indoxyl sulfate** | Amino acid | Tryptophan metabolism |
| **N-acetylglycine** | Amino acid | Glycine, serine and threonine metabolism |
| **bilirubin (Z,Z)** | Cofactors and vitamins | Hemoglobin and porphyrin metabolism |
| **creatine** | Amino acid | Creatine metabolism |
| **erythrose** | Carbohydrate | Fructose, mannose, galactose, starch, and sucrose metabolism |
| **glycerol 2-phosphate** | Xenobiotics | Chemical |
| **threonate** | Cofactors and vitamins | Ascorbate and aldarate metabolism |
| **cysteine** | Amino acid | Cysteine, methionine, SAM, taurine metabolism |
| **N-(2-furoyl)glycine** | Xenobiotics | Food component, Plant |
| **DSGEGDFXAEGGGVR*** | Peptide | Fibrinogen cleavage peptide |
| **pyridoxate** | Cofactors and vitamins | Vitamin B6 metabolism |
| **androsterone sulfate** | Lipid | Sterol, Steroid |
| **3-carboxy-4-methyl-5-propyl-2-furanpropanoate (CMPF)** | Lipid | Fatty acid, dicarboxylate |
| **3-(4-hydroxyphenyl)lactate** | Amino acid | Phenylalanine & tyrosine metabolism |
| **acetylcarnitine** | Lipid | Carnitine metabolism |
| **serine** | Amino acid | Glycine, serine and threonine metabolism |
| **trans-4-hydroxyproline** | Amino acid | Urea cycle |
| **glutamate** | Amino acid | Glutamate metabolism |
| **hexanoylcarnitine** | Lipid | Carnitine metabolism |
| **glycine** | Amino acid | Glycine, serine and threonine metabolism |
| **alanine** | Amino acid | Alanine and aspartate metabolism |
| **glycochenodeoxycholate** | Lipid | Bile acid metabolism |
| **2-aminobutyrate** | Amino acid | Butanoate metabolism |
| **scyllo-inositol** | Lipid | Inositol metabolism |
| **dodecanedioate** | Lipid | Fatty acid, dicarboxylate |
| **gamma-glutamylvaline** | Peptide | gamma-glutamyl |
| **indolepropionate** | Amino acid | Tryptophan metabolism |
| **butyrylcarnitine** | Lipid | Fatty acid metabolism (also BCAA metabolism) |
| **myristoleate (14:1n5)** | Lipid | Long chain fatty acid |
| **dehydroisoandrosterone sulfate (DHEA-S)** | Lipid | Sterol, Steroid |
| **3-methylxanthine** | Xenobiotics | Xanthine metabolism |
| **propionylcarnitine** | Lipid | Fatty acid metabolism (also BCAA metabolism) |
| **caproate (6:0)** | Lipid | Medium chain fatty acid |
| **caprylate (8:0)** | Lipid | Medium chain fatty acid |
| **10-undecenoate (11:1n1)** | Lipid | Medium chain fatty acid |
| **docosapentaenoate (n3 DPA** | Lipid | Essential fatty acid |
| **phenol sulfate** | Amino acid | Phenylalanine & tyrosine metabolism |
| **bilirubin (E,E)*** | Cofactors and vitamins | Hemoglobin and porphyrin metabolism |
| **heme*** | Cofactors and vitamins | Hemoglobin and porphyrin metabolism |
| **1-linoleoylglycerophosphoethanolamine*** | Lipid | Lysolipid |
| **3-dehydrocarnitine*** | Lipid | Carnitine metabolism |
| **pyroglutamine*** | Amino acid | Glutamate metabolism |
| **C-glycosyltryptophan*** | Amino acid | Tryptophan metabolism |
| **X-03056--N-[3-(2-Oxopyrrolidin-1-yl)propyl]acetamide** | Amino acid | Polyamine metabolism |
| **X-11422--xanthine** | Nucleotide | Purine metabolism, (hypo)xanthine/inosine containing |
| **X-11423--O-sulfo-L-tyrosine** | Amino acid | Phenylalanine & tyrosine metabolism |
| **HWESASXX*** | Peptide | Polypeptide |
| **X-11593--O-methylascorbate*** | Cofactors and vitamins | Ascorbate and aldarate metabolism |
| **adrenate (22:4n6)** | Lipid | Long chain fatty acid |
| **homostachydrine*** | Xenobiotics | Food component, Plant |
| **ADSGEGDFXAEGGGVR*** | Peptide | Fibrinogen cleavage peptide |
| **X-11793--oxidized bilirubin*** | Cofactors and vitamins | Hemoglobin and porphyrin metabolism |
| **2-hydroxyacetaminophen sulfate*** | Xenobiotics | Drug |
| **2-methoxyacetaminophen sulfate*** | Xenobiotics | Drug |
| **1-arachidonoylglycerophosphocholine*** | Lipid | Lysolipid |
| **1-palmitoleoylglycerophosphocholine*** | Lipid | Lysolipid |
| **gamma-glutamylthreonine*** | Peptide | gamma-glutamyl |
| **salicyluric glucuronide*** | Xenobiotics | Drug |
| **gamma-tocopherol** | Cofactors and vitamins | Tocopherol metabolism |
| **gamma-glutamylphenylalanine** | Peptide | gamma-glutamyl |
| **p-acetamidophenylglucuronide** | Xenobiotics | Drug |
| **isobutyrylcarnitine** | Amino acid | Valine, leucine and isoleucine metabolism |
| **pseudouridine** | Nucleotide | Pyrimidine metabolism, uracil containing |
| **valerate** | Lipid | Short chain fatty acid |
| **palmitoleate (16:1n7)** | Lipid | Long chain fatty acid |
| **alpha-ketoglutarate** | Energy | Krebs cycle |
| **erythronate*** | Carbohydrate | Aminosugars metabolism |
| **lathosterol** | Lipid | Sterol, Steroid |
| **X-12095--N1-methyl-3-pyridone-4-carboxamide** | Nucleotide | NAD metabolism |
| **X-12100--hydroxytryptophan*** | Amino acid | Tryptophan metabolism |
| **eicosenoate (20:1n9 or 11)** | Lipid | Long chain fatty acid |
| **X-12244--N-acetylcarnosine** | Peptide | Dipeptide |
| **ADpSGEGDFXAEGGGVR*** | Peptide | Fibrinogen cleavage peptide |
| **1-eicosatrienoylglycerophosphocholine*** | Lipid | Lysolipid |
| **1-docosahexaenoylglycerophosphocholine*** | Lipid | Lysolipid |
| **1-eicosadienoylglycerophosphocholine*** | Lipid | Lysolipid |
| **X-12442--5,8-tetradecadienoate** | Lipid | Long chain fatty acid |
| **piperine** | Xenobiotics | Food component, Plant |
| **octanoylcarnitine** | Lipid | Carnitine metabolism |
| **alpha-hydroxyisovalerate** | Amino acid | Valine, leucine and isoleucine metabolism |
| **N-acetylthreonine** | Amino acid | Glycine, serine and threonine metabolism |
| **decanoylcarnitine** | Lipid | Carnitine metabolism |
| **1-palmitoylglycerophosphocholine** | Lipid | Lysolipid |
| **1-heptadecanoylglycerophosphocholine** | Lipid | Lysolipid |
| **1-oleoylglycerophosphocholine** | Lipid | Lysolipid |
| **1-stearoylglycerophosphocholine** | Lipid | Lysolipid |
| **5-dodecenoate (12:1n7)** | Lipid | Medium chain fatty acid |
| **stearidonate (18:4n3)** | Lipid | Long chain fatty acid |
| **10-heptadecenoate (17:1n7)** | Lipid | Long chain fatty acid |
| **10-nonadecenoate (19:1n9)** | Lipid | Long chain fatty acid |
| **epiandrosterone sulfate** | Lipid | Sterol, Steroid |
| **linolenate [alpha or gamma** | Lipid | Essential fatty acid |
| **X-12510--2-aminooctanoic acid** | Amino acid | Amino fatty acid |
| **bilirubin (E,Z or Z,E)*** | Cofactors and vitamins | Hemoglobin and porphyrin metabolism |
| **metoprolol acid metabolite*** | Xenobiotics | Drug |
| **1-arachidonoylglycerophosphoinositol*** | Lipid | Lysolipid |
| **asparagine** | Amino acid | Alanine and aspartate metabolism |
| **3-(cystein-S-yl)acetaminophen*** | Xenobiotics | Drug |
| **stachydrine** | Xenobiotics | Food component, Plant |
| **1-methylxanthine** | Xenobiotics | Xanthine metabolism |
| **7-methylxanthine** | Xenobiotics | Xanthine metabolism |
| **1-methylurate** | Xenobiotics | Xanthine metabolism |
| **1,7-dimethylurate** | Xenobiotics | Xanthine metabolism |
| **1,3,7-trimethylurate** | Xenobiotics | Xanthine metabolism |
| **isovalerylcarnitine** | Amino acid | Valine, leucine and isoleucine metabolism |
| **stearoylcarnitine** | Lipid | Carnitine metabolism |
| **1-stearoylglycerophosphoethanolamine** | Lipid | Lysolipid |
| **1-linoleoylglycerophosphocholine** | Lipid | Lysolipid |
| **bradykinin, des-arg(9)** | Peptide | Polypeptide |
| **gamma-glutamylisoleucine*** | Peptide | gamma-glutamyl |
| **laurylcarnitine** | Lipid | Carnitine metabolism |
| **isovalerate** | Lipid | Fatty acid metabolism |
| **7-methylguanine** | Nucleotide | Purine metabolism, guanine containing |
| **phenylacetylglutamine** | Amino acid | Phenylalanine & tyrosine metabolism |
| **pro-hydroxy-pro** | Peptide | Dipeptide |
| **N2,N2-dimethylguanosine** | Nucleotide | Purine metabolism, guanine containing |
| **oleoylcarnitine** | Lipid | Carnitine metabolism |
| **1-arachidonoylglycerophosphoethanolamine*** | Lipid | Lysolipid |
| **X-13431--nonanoylcarnitine*** | Lipid | Carnitine metabolism |
| **2-palmitoylglycerophosphocholine*** | Lipid | Lysolipid |
| **2-oleoylglycerophosphocholine*** | Lipid | Lysolipid |
| **2-stearoylglycerophosphocholine*** | Lipid | Lysolipid |
| **2-linoleoylglycerophosphocholine*** | Lipid | Lysolipid |
| **1-palmitoylglycerophosphoinositol*** | Lipid | Lysolipid |
| **catechol sulfate** | Xenobiotics | Benzoate metabolism |
| **2-methylbutyroylcarnitine** | Amino acid | Valine, leucine and isoleucine metabolism |
| **hydroxyisovaleroyl carnitine** | Amino acid | Valine, leucine and isoleucine metabolism |
| **glutaroyl carnitine** | Amino acid | Lysine metabolism |
| **2-tetradecenoyl carnitine** | Lipid | Carnitine metabolism |
| **4-hydroxyhippurate** | Xenobiotics | Benzoate metabolism |
| **1-myristoylglycerophosphocholine** | Lipid | Lysolipid |
| **1-oleoylglycerophosphoethanolamine** | Lipid | Lysolipid |
| **1-palmitoylglycerophosphoethanolamine** | Lipid | Lysolipid |
| **3-(3-hydroxyphenyl)propionate** | Amino acid | Phenylalanine & tyrosine metabolism |
| **tetradecanedioate** | Lipid | Fatty acid, dicarboxylate |
| **2-hydroxypalmitate** | Lipid | Fatty acid, monohydroxy |
| **hexadecanedioate** | Lipid | Fatty acid, dicarboxylate |
| **dihomo-linolenate (20:3n3 or n6)** | Lipid | Essential fatty acid |
| **threitol** | Carbohydrate | Nucleotide sugars, pentose metabolism |
| **thymol sulfate** | Xenobiotics | Food component, Plant |
| **4-vinylphenol sulfate** | Xenobiotics | Benzoate metabolism |
| **4-ethylphenylsulfate** | Xenobiotics | Benzoate metabolism |
| **p-cresol sulfate** | Amino acid | Phenylalanine & tyrosine metabolism |
| **octadecanedioate** | Lipid | Fatty acid, dicarboxylate |
| **leucylleucine** | Peptide | Dipeptide |
| **7-alpha-hydroxy-3-oxo-4-cholestenoate (7-Hoca)** | Lipid | Sterol, Steroid |
| **n-Butyl Oleate** | Lipid | Fatty acid, ester |
| **dimethylarginine (SDMA + ADMA)** | Amino acid | Urea cycle |
| **taurolithocholate 3-sulfate** | Lipid | Bile acid metabolism |
| **carbamazepine*** | Xenobiotics | Drug |
| **succinylcarnitine** | Energy | Krebs cycle |
| **tryptophan betaine** | Amino acid | Tryptophan metabolism |
| **cyclo(leu-pro)** | Peptide | Dipeptide |
| **chiro-inositol** | Lipid | Inositol metabolism |
| **5alpha-androstan-3beta,17beta-diol disulfate** | Lipid | Sterol, Steroid |
| **5alpha-pregnan-3beta,20alpha-diol disulfate** | Lipid | Sterol, Steroid |
| **4-androsten-3beta,17beta-diol disulfate 1*** | Lipid | Sterol, Steroid |
| **4-androsten-3beta,17beta-diol disulfate 2*** | Lipid | Sterol, Steroid |
| **2-hydroxyglutarate** | Lipid | Fatty acid, dicarboxylate |
| **ergothioneine** | Xenobiotics | Food component, Plant |
| **palmitoyl sphingomyelin** | Lipid | Sphingolipid |
| **phenylalanylphenylalanine** | Peptide | Dipeptide |
| **cis-4-decenoyl carnitine** | Lipid | Carnitine metabolism |
| **atenolol** | Xenobiotics | Drug |
| **15-methylpalmitate (isobar with 2-methylpalmitate)** | Lipid | Fatty acid, branched |
| **1-palmitoylplasmenylethanolamine*** | Lipid | Lysolipid |
| **glycoursodeoxycholate** | Lipid | Bile acid metabolism |

**Supplementary Table S7: Associations between metabolite and metabolite batch**

| **Metabolite Name** | **Beta** | **SE** | **P** |
| --- | --- | --- | --- |
| **1-arachidonoylglycerophosphocholine*** | -0.0071708 | 0.0225002 | NS |
| **1-arachidonoylglycerophosphoethanolamine*** | 0.0186646 | 0.0217021 | NS |
| **1-arachidonoylglycerophosphoinositol*** | 0.0985834 | 0.0199041 | 7.70E-07 |
| **1-docosahexaenoylglycerophosphocholine*** | 0.0864364 | 0.0197311 | 1.22E-05 |
| **1-eicosadienoylglycerophosphocholine*** | -0.0468758 | 0.0215032 | 0.029 |
| **1-eicosatrienoylglycerophosphocholine*** | -0.0233715 | 0.0208744 | NS |
| **1-heptadecanoylglycerophosphocholine** | -0.0042177 | 0.0201866 | NS |
| **1-linoleoylglycerophosphocholine** | -0.0922986 | 0.0206516 | 8.13E-06 |
| **1-linoleoylglycerophosphoethanolamine*** | -0.033525 | 0.0216038 | NS |
| **1-methylurate** | 0.0544171 | 0.0233023 | 0.0120 |
| **1-methylxanthine** | -0.0003479 | 0.0230508 | NS |
| **1-myristoylglycerophosphocholine** | 0.0086544 | 0.0205496 | NS |
| **1-oleoylglycerol (1-monoolein)** | 0.0445629 | 0.0235933 | NS |
| **1-oleoylglycerophosphocholine** | -0.0402309 | 0.0195465 | 0.040 |
| **1-oleoylglycerophosphoethanolamine** | -0.0147206 | 0.0202513 | NS |
| **1-palmitoleoylglycerophosphocholine*** | 0.0274586 | 0.021126 | NS |
| **1-palmitoylglycerol (1-monopalmitin)** | 0.0840785 | 0.0202976 | 3.53E-05 |
| **1-palmitoylglycerophosphocholine** | -0.0244669 | 0.0199171 | NS |
| **1-palmitoylglycerophosphoethanolamine** | 0.0814648 | 0.0198069 | 4.01E-05 |
| **1-palmitoylglycerophosphoinositol*** | 0.0468286 | 0.0215139 | 0.030 |
| **1-palmitoylplasmenylethanolamine*** | 0.0780345 | 0.0339375 | 0.022 |
| **1-stearoylglycerol (1-monostearin)** | 0.0924559 | 0.020696 | 8.21E-06 |
| **1-stearoylglycerophosphocholine** | -0.0072175 | 0.0175695 | NS |
| **1-stearoylglycerophosphoethanolamine** | 0.0802484 | 0.0205914 | 9.94E-05 |
| **1-stearoylglycerophosphoinositol** | 0.0726559 | 0.0215783 | 7.69E-04 |
| **1,3,7-trimethylurate** | 0.0669913 | 0.0287973 | 0.020 |
| **1,5-anhydroglucitol (1,5-AG)** | -0.092879 | 0.0219369 | 2.36E-05 |
| **1,6-anhydroglucose** | 0.0520616 | 0.0298312 | NS |
| **1,7-dimethylurate** | 0.1225956 | 0.0223231 | 4.32E-08 |
| **10-heptadecenoate (17:1n7)** | 0.1080396 | 0.0218276 | 7.83E-07 |
| **10-nonadecenoate (19:1n9)** | 0.0927705 | 0.0219502 | 2.44E-05 |
| **10-undecenoate (11:1n1)** | 0.0260866 | 0.0214968 | NS |
| **15-methylpalmitate (isobar with 2-methylpalmitate)** | 0.2586345 | 0.0366656 | 2.22E-12 |
| **2-aminobutyrate** | 0.035507 | 0.0204114 | NS |
| **2-hydroxyacetaminophen sulfate*** | 0.0585774 | 0.0389746 | NS |
| **2-hydroxybutyrate (AHB)** | 0.0916561 | 0.0204554 | 7.71E-06 |
| **2-hydroxyglutarate** | 0.0564051 | 0.0363371 | NS |
| **2-hydroxyhippurate (salicylurate)** | 0.0278888 | 0.0329472 | NS |
| **2-hydroxyisobutyrate** | 0.0533406 | 0.0202032 | 0.008 |
| **2-hydroxypalmitate** | 0.0433092 | 0.0218263 | 0.047 |
| **2-hydroxystearate** | 0.0560569 | 0.0218028 | 0.010 |
| **2-linoleoylglycerophosphocholine*** | -0.0982919 | 0.0191605 | 3.09E-07 |
| **2-methoxyacetaminophen sulfate*** | 0.108899 | 0.0472136 | 0.021 |
| **2-methylbutyroylcarnitine** | 0.0418931 | 0.019767 | 0.034 |
| **2-oleoylglycerophosphocholine*** | -0.0426334 | 0.019931 | 0.033 |
| **2-palmitoylglycerophosphocholine*** | -0.0349174 | 0.0208465 | NS |
| **2-stearoylglycerophosphocholine*** | -0.0110269 | 0.0192645 | NS |
| **2-tetradecenoyl carnitine** | 0.0407551 | 0.022333 | NS |
| **3-(3-hydroxyphenyl)propionate** | -0.0043849 | 0.0516934 | NS |
| **3-(4-hydroxyphenyl)lactate** | -0.0017094 | 0.0210127 | NS |
| **3-(cystein-S-yl)acetaminophen*** | 0.0785502 | 0.0470748 | NS |
| **3-carboxy-4-methyl-5-propyl-2-furanpropanoate (CMPF)** | 0.1794105 | 0.0201921 | 1.06E-18 |
| **3-dehydrocarnitine*** | 0.1108847 | 0.0202957 | 5.04E-08 |
| **3-hydroxybutyrate (BHBA)** | 0.073828 | 0.0207339 | 3.75E-04 |
| **3-indoxyl sulfate** | 0.0687483 | 0.0197348 | 5.016E-04 |
| **3-methoxytyrosine** | -0.0279384 | 0.0250611 | NS |
| **3-methyl-2-oxobutyrate** | -0.0425088 | 0.0238939 | NS |
| **3-methyl-2-oxovalerate** | -0.1201695 | 0.0230892 | 2.07E-07 |
| **3-methylhistidine** | 0.0078202 | 0.0217479 | NS |
| **3-methylxanthine** | 0.0010419 | 0.0239862 | NS |
| **3-phenylpropionate (hydrocinnamate)** | -0.0358969 | 0.0217608 | NS |
| **4-acetamidobutanoate** | 0.1184282 | 0.021384 | 3.33E-08 |
| **4-acetamidophenol** | 0.062205 | 0.0539883 | NS |
| **4-androsten-3beta,17beta-diol disulfate 1*** | -0.303317 | 0.0344019 | 2.05E-18 |
| **4-androsten-3beta,17beta-diol disulfate 2*** | -0.4046282 | 0.0332125 | 2.76E-33 |
| **4-ethylphenylsulfate** | 0.0023753 | 0.0218957 | NS |
| **4-hydroxyhippurate** | 0.078295 | 0.0375287 | 0.037 |
| **4-methyl-2-oxopentanoate** | -0.1162656 | 0.0230818 | 5.00E-07 |
| **4-vinylphenol sulfate** | -0.017548 | 0.0220348 | NS |
| **5-dodecenoate (12:1n7)** | 0.0795019 | 0.0218725 | 2.83E-04 |
| **5-oxoproline** | 0.0135755 | 0.019534 | NS |
| **5alpha-androstan-3beta,17beta-diol disulfate** | -0.3305273 | 0.0358145 | 5.46E-20 |
| **5alpha-pregnan-3beta,20alpha-diol disulfate** | -0.2517194 | 0.035075 | 9.39E-13 |
| **7-alpha-hydroxy-3-oxo-4-cholestenoate (7-Hoca)** | 0.0387077 | 0.0201011 | NS |
| **7-methylguanine** | -0.0304519 | 0.0189511 | NS |
| **7-methylxanthine** | -0.0388153 | 0.0234388 | NS |
| **acetylcarnitine** | 0.1380536 | 0.0217202 | 2.38E-10 |
| **acetylphosphate** | 0.0929751 | 0.0190422 | 1.10E-06 |
| **ADpSGEGDFXAEGGGVR*** | 0.0867468 | 0.0275822 | 0.002 |
| **adrenate (22:4n6)** | 0.0778916 | 0.0200899 | 1.08E-04 |
| **ADSGEGDFXAEGGGVR*** | 0.0456732 | 0.0187874 | 0.015 |
| **alanine** | 0.0477267 | 0.020155 | 0.018 |
| **allantoin** | 0.037917 | 0.0231454 | NS |
| **alpha-hydroxyisovalerate** | -0.0924548 | 0.0211571 | 1.284E-05 |
| **alpha-ketoglutarate** | 0.0540087 | 0.0255076 | 0.034 |
| **alpha-tocopherol** | 0.0869448 | 0.0217615 | 6.61E-05 |
| **androsterone sulfate** | -0.1715267 | 0.0204732 | 8.06E-17 |
| **arabinose** | 0.1132301 | 0.023861 | 2.18E-06 |
| **arachidonate (20:4n6)** | 0.0501691 | 0.0222189 | 0.024 |
| **arginine** | -0.0226034 | 0.0188518 | NS |
| **asparagine** | -0.0484479 | 0.0195053 | 0.013 |
| **aspartate** | -0.0190226 | 0.020845 | NS |
| **aspartylphenylalanine** | 0.0349512 | 0.0268957 | NS |
| **atenolol** | 0.0882454 | 0.1430192 | NS |
| **benzoate** | 0.0684459 | 0.0207904 | 0.001 |
| **beta-hydroxyisovalerate** | 0.0360091 | 0.0210813 | NS |
| **betaine** | -0.0043749 | 0.0205096 | NS |
| **bilirubin (E,E)*** | -0.0208011 | 0.0213062 | NS |
| **bilirubin (E,Z or Z,E)*** | -0.0414065 | 0.0250355 | NS |
| **bilirubin (Z,Z)** | -0.0355483 | 0.0219376 | NS |
| **biliverdin** | -0.0489321 | 0.0248121 | 0.049 |
| **bradykinin, des-arg(9)** | -0.025532 | 0.0225345 | NS |
| **butyrylcarnitine** | 0.0912336 | 0.0216766 | 2.64E-05 |
| **C-glycosyltryptophan*** | 0.2211126 | 0.0192925 | 8.24E-30 |
| **caffeine** | 0.0984934 | 0.0206994 | 2.04E-06 |
| **caprate (10:0)** | 0.0550202 | 0.0196066 | 0.005 |
| **caproate (6:0)** | 0.0614904 | 0.0219377 | 0.005 |
| **caprylate (8:0)** | 0.0770076 | 0.020238 | 1.44E-04 |
| **carbamazepine*** | -0.1235261 | 0.1116648 | NS |
| **carnitine** | 0.0289068 | 0.0198768 | NS |
| **catechol sulfate** | 0.0290613 | 0.0210557 | NS |
| **chiro-inositol** | 0.0003368 | 0.0534589 | NS |
| **cholate** | 0.0751386 | 0.0223724 | 7.94E-04 |
| **cholesterol** | 0.1504076 | 0.0200199 | 7.53E-14 |
| **choline** | 0.0777078 | 0.0198375 | 9.15E-05 |
| **cis-4-decenoyl carnitine** | 0.1362541 | 0.0366796 | 2.08E-04 |
| **citrate** | 0.1167343 | 0.0207638 | 2.06E-08 |
| **citrulline** | 0.1061766 | 0.0200997 | 1.36E-07 |
| **cortisol** | 0.0011655 | 0.0198865 | NS |
| **cortisone** | -0.0839829 | 0.0208807 | 5.91E-05 |
| **cotinine** | 0.0138504 | 0.0460623 | NS |
| **creatine** | 0.1104318 | 0.0201183 | 4.37E-08 |
| **creatinine** | -0.0700567 | 0.0212913 | 0.001 |
| **cyclo(leu-pro)** | -0.0444484 | 0.0381087 | NS |
| **cysteine** | 0.0329556 | 0.0207196 | NS |
| **decanoylcarnitine** | 0.0389766 | 0.0208828 | NS |
| **dehydroisoandrosterone sulfate (DHEA-S)** | -0.2465216 | 0.01846 | 1.38E-39 |
| **deoxycholate** | 0.0308564 | 0.0221036 | NS |
| **dihomo-linoleate (20:2n6)** | 0.0498778 | 0.0217261 | 0.022 |
| **dihomo-linolenate (20:3n3 or n6)** | 0.0422442 | 0.0210021 | 0.044 |
| **dimethylarginine (SDMA + ADMA)** | 0.0270679 | 0.0204807 | NS |
| **docosahexaenoate (DHA** | 0.1225789 | 0.020784 | 4.08E-09 |
| **docosapentaenoate (n3 DPA** | 0.1354946 | 0.0214726 | 3.19E-10 |
| **dodecanedioate** | 0.0625021 | 0.0212646 | 0.003 |
| **DSGEGDFXAEGGGVR*** | 0.0876672 | 0.0231523 | 1.57E-04 |
| **eicosapentaenoate (EPA** | 0.1748933 | 0.0211522 | 2.00E-16 |
| **eicosenoate (20:1n9 or 11)** | 0.0804276 | 0.0213426 | 1.67E-04 |
| **epiandrosterone sulfate** | -0.175558 | 0.0203022 | 8.35E-18 |
| **ergothioneine** | -0.0758034 | 0.0426119 | NS |
| **erythritol** | 0.1824578 | 0.0200856 | 1.82E-19 |
| **erythronate*** | 0.0685757 | 0.0210668 | 0.001 |
| **erythrose** | 0.0220926 | 0.0212656 | NS |
| **estrone 3-sulfate** | 0.0322388 | 0.0567806 | NS |
| **fructose** | 0.0093471 | 0.0218934 | NS |
| **gamma-glutamylglutamine** | 0.046888 | 0.018838 | 0.013 |
| **gamma-glutamylisoleucine*** | 0.0463818 | 0.0271984 | NS |
| **gamma-glutamylleucine** | 0.0352816 | 0.021764 | NS |
| **gamma-glutamylphenylalanine** | 0.1068344 | 0.0208432 | 3.15E-07 |
| **gamma-glutamylthreonine*** | -0.0866631 | 0.0254793 | 6.85E-04 |
| **gamma-glutamyltyrosine** | 0.1199104 | 0.0209619 | 1.17E-08 |
| **gamma-glutamylvaline** | 0.0546741 | 0.0214276 | 0.011 |
| **gamma-tocopherol** | 0.0151 | 0.022353 | NS |
| **glucose** | 0.0887757 | 0.019292 | 4.36E-06 |
| **glutamate** | 0.0934465 | 0.019039 | 9.67E-07 |
| **glutamine** | 0.0355387 | 0.0204514 | NS |
| **glutaroyl carnitine** | 0.0294994 | 0.0217561 | NS |
| **glycerate** | 0.0027165 | 0.0206285 | NS |
| **glycerol** | 0.1396092 | 0.021622 | 1.24E-10 |
| **glycerol 2-phosphate** | 0.0112128 | 0.0231912 | NS |
| **glycerol 3-phosphate (G3P)** | 0.0721291 | 0.0204035 | 4.14E-04 |
| **glycerophosphorylcholine (GPC)** | 0.0193033 | 0.0210711 | NS |
| **glycine** | 0.0143708 | 0.0202598 | NS |
| **glycochenodeoxycholate** | 0.0149088 | 0.0222169 | NS |
| **glycocholate** | -0.0088329 | 0.023474 | NS |
| **glycoursodeoxycholate** | -0.0551502 | 0.0362527 | NS |
| **heme*** | -0.0850166 | 0.0249605 | 6.69E-04 |
| **heptanoate (7:0)** | 0.0724787 | 0.0206464 | 4.54E-04 |
| **hexadecanedioate** | 0.1203511 | 0.0224951 | 9.44E-08 |
| **hexanoylcarnitine** | 0.1391508 | 0.020216 | 7.06E-12 |
| **hippurate** | 0.08444 | 0.0197622 | 1.99E-05 |
| **histidine** | -0.0843089 | 0.0214591 | 8.72E-05 |
| **homocitrulline** | 0.0847901 | 0.0262025 | 0.001 |
| **homostachydrine*** | 0.0288139 | 0.0409826 | NS |
| **HWESASXX*** | 0.0552866 | 0.0226981 | 0.015 |
| **hydroxyisovaleroyl carnitine** | 0.0291716 | 0.021857 | NS |
| **hyodeoxycholate** | 0.0606202 | 0.0235634 | 0.010 |
| **hypoxanthine** | -0.0162761 | 0.0209752 | NS |
| **ibuprofen** | 0.022662 | 0.0479896 | NS |
| **indoleacetate** | 0.0177068 | 0.0201323 | NS |
| **indolelactate** | -0.0452612 | 0.0205689 | 0.028 |
| **indolepropionate** | -0.0659229 | 0.0199307 | 9.52E-04 |
| **isobutyrylcarnitine** | 0.0522161 | 0.0212271 | 0.014 |
| **isoleucine** | -0.0336424 | 0.0217678 | NS |
| **isovalerate** | -0.0122455 | 0.023229 | NS |
| **isovalerylcarnitine** | 0.0171986 | 0.0203325 | NS |
| **kynurenine** | 0.0944371 | 0.0205225 | 4.36E-06 |
| **lactate** | -0.0189102 | 0.0218241 | NS |
| **lathosterol** | 0.0294477 | 0.0240861 | NS |
| **laurate (12:0)** | 0.080555 | 0.0207795 | 1.08E-04 |
| **laurylcarnitine** | 0.0146611 | 0.0241592 | NS |
| **leucine** | -0.0100797 | 0.0215166 | NS |
| **leucylleucine** | -0.0089704 | 0.0256744 | NS |
| **levulinate (4-oxovalerate)** | 0.0254991 | 0.0207537 | NS |
| **linoleate (18:2n6)** | 0.067759 | 0.0221235 | 0.002 |
| **linolenate [alpha or gamma** | 0.0449957 | 0.0219444 | 0.040 |
| **lysine** | 0.0275098 | 0.0203289 | NS |
| **malate** | 0.0667494 | 0.0219933 | 0.002 |
| **mannitol** | 0.0696327 | 0.023228 | 0.003 |
| **mannose** | 0.069213 | 0.0195091 | 3.94E-04 |
| **margarate (17:0)** | 0.1093238 | 0.0204826 | 1.01E-07 |
| **methionine** | -0.07038 | 0.02265 | 0.002 |
| **metoprolol acid metabolite*** | 0.0539548 | 0.0972402 | NS |
| **myo-inositol** | 0.1113976 | 0.018968 | 4.74E-09 |
| **myristate (14:0)** | 0.0957771 | 0.0220052 | 1.39E-05 |
| **myristoleate (14:1n5)** | 0.0897932 | 0.0215604 | 3.20E-05 |
| **N-(2-furoyl)glycine** | -0.0445767 | 0.1049479 | NS |
| **N-acetylalanine** | 0.1124081 | 0.0202167 | 2.93E-08 |
| **N-acetylglycine** | 0.0485566 | 0.0211048 | 0.021 |
| **N-acetylornithine** | 0.0140686 | 0.0207716 | NS |
| **N-acetylthreonine** | 0.0457512 | 0.0209327 | 0.029 |
| **n-Butyl Oleate** | 0.0587831 | 0.0239543 | 0.014 |
| **N1-methyladenosine** | 0.0982227 | 0.0203542 | 1.46E-06 |
| **N2,N2-dimethylguanosine** | 0.126124 | 0.0270913 | 3.40E-06 |
| **naproxen** | 0.029454 | 0.2077179 | NS |
| **nonadecanoate (19:0)** | 0.0759016 | 0.0213706 | 3.89E-04 |
| **octadecanedioate** | 0.0454589 | 0.0208449 | 0.030 |
| **octanoylcarnitine** | 0.1094491 | 0.020923 | 1.80E-07 |
| **oleate (18:1n9)** | 0.0761983 | 0.0219004 | 5.10E-04 |
| **oleoylcarnitine** | 0.0061067 | 0.0218629 | NS |
| **ornithine** | -0.0209146 | 0.0229111 | NS |
| **p-acetamidophenylglucuronide** | 0.1498607 | 0.0404504 | 2.22E-04 |
| **p-cresol sulfate** | 0.099941 | 0.019455 | 2.96E-07 |
| **palmitate (16:0)** | 0.1028066 | 0.0220044 | 3.11E-06 |
| **palmitoleate (16:1n7)** | 0.0885402 | 0.0217275 | 4.72E-05 |
| **palmitoyl sphingomyelin** | 0.2727107 | 0.0338292 | 1.12E-15 |
| **palmitoylcarnitine** | 0.0231638 | 0.021013 | NS |
| **pantothenate** | 0.0688479 | 0.0201724 | 6.51E-04 |
| **paraxanthine** | 0.0770216 | 0.0205762 | 1.85E-04 |
| **pelargonate (9:0)** | 0.0753032 | 0.0195997 | 1.24E-04 |
| **pentadecanoate (15:0)** | 0.0996301 | 0.0207058 | 1.57E-06 |
| **phenol sulfate** | 0.0627097 | 0.0197354 | 0.002 |
| **phenylacetate** | 0.0057394 | 0.0239162 | NS |
| **phenylacetylglutamine** | 0.1026987 | 0.0203741 | 4.91E-07 |
| **phenylalanine** | 0.0689186 | 0.0225604 | 0.002 |
| **phenylalanylphenylalanine** | -0.0666335 | 0.0497477 | NS |
| **phenyllactate (PLA)** | -0.0735278 | 0.0237432 | 0.002 |
| **phosphate** | 0.0711499 | 0.0227055 | 0.002 |
| **pipecolate** | -0.0837023 | 0.0197074 | 2.23E-05 |
| **piperine** | -0.0354201 | 0.0212813 | NS |
| **pro-hydroxy-pro** | 0.0681278 | 0.0198271 | 5.98 |
| **proline** | 7.63E-07 | 0.0222112 | NS |
| **propionylcarnitine** | 0.1064936 | 0.0207282 | 2.96E-07 |
| **pseudouridine** | 0.1143033 | 0.0204361 | 2.42E-08 |
| **pyridoxate** | 0.0414007 | 0.0194045 | 0.033 |
| **pyroglutamine*** | -0.0556365 | 0.0213379 | 0.009 |
| **pyruvate** | -0.0338728 | 0.0226383 | NS |
| **quinate** | 0.0490516 | 0.0228988 | 0.032 |
| **saccharin** | 0.0045574 | 0.0340808 | NS |
| **salicylate** | 0.0979666 | 0.0394138 | 0.013 |
| **salicyluric glucuronide*** | 0.0775899 | 0.0556325 | NS |
| **scyllo-inositol** | 0.0520722 | 0.0218434 | 0.017 |
| **serine** | -0.0081289 | 0.0203271 | NS |
| **serotonin (5HT)** | 0.0445344 | 0.0217316 | 0.041 |
| **stachydrine** | -0.0198114 | 0.0200725 | NS |
| **stearate (18:0)** | 0.0955624 | 0.0219306 | 1.36E-05 |
| **stearidonate (18:4n3)** | 0.0887542 | 0.0213196 | 3.23E-05 |
| **stearoylcarnitine** | 0.0275689 | 0.0214467 | NS |
| **succinylcarnitine** | 0.1074245 | 0.0215125 | 6.27E-07 |
| **taurochenodeoxycholate** | -0.0160111 | 0.0240782 | NS |
| **taurocholate** | -0.0054765 | 0.0266633 | NS |
| **taurolithocholate 3-sulfate** | 0.0117145 | 0.0207291 | NS |
| **tetradecanedioate** | 0.0997549 | 0.025972 | 1.25E-04 |
| **theobromine** | -0.003984 | 0.0204015 | NS |
| **theophylline** | 0.071232 | 0.0206354 | 5.64E-04 |
| **threitol** | 0.1622272 | 0.0201975 | 1.36E-15 |
| **threonate** | -0.0578887 | 0.0221969 | 0.009 |
| **threonine** | -0.0711635 | 0.0210391 | 7.29E-04 |
| **thymol sulfate** | -0.0195428 | 0.0270332 | NS |
| **trans-4-hydroxyproline** | 0.0136023 | 0.0212563 | NS |
| **tryptophan** | -0.0849671 | 0.0217167 | 9.33E-05 |
| **tryptophan betaine** | -0.112753 | 0.0359939 | 0.002 |
| **tyrosine** | 0.0692554 | 0.0212734 | 1.14E-04 |
| **undecanoate (11:0)** | 0.0555992 | 0.0180123 | 0.002 |
| **urate** | 0.0535263 | 0.0193161 | 0.006 |
| **urea** | 0.1026074 | 0.0192294 | 1.02E-07 |
| **uridine** | -0.0208668 | 0.0218247 | NS |
| **ursodeoxycholate** | 0.0007103 | 0.0223807 | NS |
| **valerate** | 0.0192786 | 0.0242755 | NS |
| **valine** | 0.0043588 | 0.0211783 | NS |
| **X-03056--N-[3-(2-Oxopyrrolidin-1-yl)propyl]acetamide** | 0.0458571 | 0.0211774 | 0.030 |
| **X-04499--3,4-dihydroxybutyrate** | 0.0952466 | 0.0226118 | 2.60E-05 |
| **X-11422--xanthine** | 0.0192785 | 0.0203858 | NS |
| **X-11423--O-sulfo-L-tyrosine** | 0.1012158 | 0.0209745 | 1.46E-06 |
| **X-11593--O-methylascorbate*** | 0.14225 | 0.0193302 | 2.37E-13 |
| **X-11793--oxidized bilirubin*** | -0.019197 | 0.0230208 | NS |
| **X-12095--N1-methyl-3-pyridone-4-carboxamide** | 0.0665004 | 0.0191549 | 5.24E-04 |
| **X-12100--hydroxytryptophan*** | 0.0622356 | 0.0207592 | 0.003 |
| **X-12244--N-acetylcarnosine** | -0.0728536 | 0.0188674 | 1.15E-04 |
| **X-12442--5,8-tetradecadienoate** | 0.0574288 | 0.0219357 | 0.009 |
| **X-12510--2-aminooctanoic acid** | -0.0731687 | 0.021737 | 7.72E-04 |
| **X-13431--nonanoylcarnitine*** | -0.0162675 | 0.0229294 | NS |
| **xanthine** | 0.0161871 | 0.0168544 | NS |

**Notes: Results of a linear regression showing the prediction of metabolite level by batch (batches 1, 2 and 3) adjusted for age, BMI and family relatedness.**

**Supplementary Text S3: Metabolic syndrome status and phenotypes**

Whole-body DXA scans (Hologic QDR; Hologic, Inc., Waltham, MA, USA), undertaken in the supine position were used to estimate waist circumference. Systolic and diastolic blood pressure (SBP and DBP, respectively) were carried out using the Marshall mb02 or the Omron Mx3 Digital Blood Pressure Monitors. An average of three readings (separated by one minute) carried out on twins in a seated position were used for analysis. Three devices (Cobas Fara; Roche Diagnostics, Lewes, UK; Kodak Ektachem dry chemistry analysers (Johnson and Johnson Vitros Ektachem machine, Beckman LX20 analysers, Roche P800 modular system)) were used to measure serum levels of total cholesterol, high density lipoprotein (HDL) cholesterol and triglycerides (TG) on fasted blood samples using enzymatic colorimetric assays. Using an enzymatic colorimetric slide assay, fasting blood glucose was measured on an Ektachem 700 multichannel analyser (Johnson and Johnson Clinical Diagnostic Systems, Amersham, UK). Twins who had at least three of any of the following factors were considered to have MetS:

1. Increased waist circumference: men ≥94 cm and women ≥80 cm.
2. Increased triglycerides (TG): >1·7 mmol/L, or treatment for abnormality
3. Lower HDL-cholesterol: men <1·03 mmol/L in men and women <1·29 mmol/L, or treatment for abnormality
4. High blood pressure: ≥130/85 mmHg, or treatment for hypertension
5. Raised fasting plasma glucose (FBG): FBG ≥ 5·6 mmol/L. Most individuals with type 2 diabetes will have metabolic syndrome based on these criteria

**Supplementary Text S4: Heritability analysis of the hippurate diet score**

The diet score heritability was estimated by linear structural equation modeling in Mx (Neale & Cardon, 1992; Neale et al., 2003). Using univariate ACE modelling, the phenotypic variance is decomposed into: A, the additive genetic effects component; C, common environmental effects component; and E, non-shared environmental effects component. In the case that MZ twins have significantly greater phenotypic similarity than DZ twins, this suggests additive genetic effects are high. The common environmental component estimates the influence of the family environment, which is assumed by the model to be equal for both MZ and DZ pairs (Kyvik, 2000). Effects for each individual are indicated by the non-shared environmental component, which also includes the measurement error. The proportion of the phenotypic variance assigned to genetic factors represents heritability, which is calculated as follows: *h^2^* = (A)/(A + C + E). Each of the ACE, AE, CE, and E models were tested and the best-fitting model evaluated and indicated by the lowest Akaike’s information criterion (AIC) (Neale & Cardon, 1992). The diet score was residual-adjusted for age and sex before analysis.

References

Kyvik, K. (2000). Generalisability and assumptions of twin studies. In T. D. Spector, H. Snieder & A. J. MacGregor (Eds.), Advances in twin and sib-pair analysis. (pp. 67–77). London: Greenwich Medical Media.

Neale, M. C., Boker, S. M., Xie, G., & Maes, H. (2003). Mx: Statistical modeling. Richmond: Department of Psychiatry, Medical College of Virginia.

Neale, M. C., & Cardon, L. R. (Eds.). (1992). Methodology for genetic studies of twins and families (Nato ISI Series D: Behavioural and Social Sciences, vol. 67). Dordrecht: Kluwer Academic Publishers

|  | **Hippurate** | |  | **Hippurate diet ^(2)^** | |
| --- | --- | --- | --- | --- | --- |
| **Level** | **Number of variables** | ***P*** |  | **Number of variables** | ***P*** |
| Phylum | 34 | 1.47x10^-3^ |  | 1 | 0.05 |
| Class | **72** | 6.94x10^-4^ |  | 2 | 0.025 |
| Order | 137 | 3.65x10^-4^ |  | 3 | 0.017 |
| Family | 194 | 2.58x10^-4^ |  | 3 | 0.017 |
| Genus | 382 | 1.31x10^-4^ |  | 7 | 7.14x10^-3^ |
| OTU | 581 | 8.61x10^-5^ |  | 30 | 1.67x10^-3^ |

**Supplementary Table S8. Statistical significance thresholds for microbiome analysis using Bonferroni correction ^(1)^**

- 1. Bonferroni correction was calculated within each level.
  2. Variables significantly associated with hippurate were tested for their association against the hippurate diet score.

**Supplementary Text S5. Metabolite trajectory association with MetS (and components) mediated by Shannon diversity, the diet score and specific taxa**

The proportion of the variance of the MetS status and its components were determined for the hippurate trajectory after taking into account all covariates. This quantity is indicated as r^2^_x_. The proportion of the variance for the MetS status (and its components) explained by the hippurate trajectory was then calculated after taking into account the same covariates as above but also including Shannon diversity, the hippurate diet score (if applicable) and associated taxa (r^2^_xy_). The percentage of the longitudinal hippurate association mediated by Shannon diversity, the hippurate diet score and associated taxa (r^2^_y_) was calculated as the proportion of the variance of MetS status (and components) that is due to the longitudinal hippurate trajectory association with Shannon diversity, the hippurate diet score and associated taxa, namely 1 – (r^2^_xy_/r^2^_x_).
